# Common population codes produce extremely nonlinear neural manifolds

**DOI:** 10.1073/pnas.2305853120

**Published:** 2023-09-21

**Authors:** Anandita De, Rishidev Chaudhuri

**Affiliations:** ^a^Center for Neuroscience, University of California, Davis, CA 95618; ^b^Department of Physics, University of California, Davis, CA 95616; ^c^Department of Neurobiology, Physiology and Behavior, University of California, Davis, CA 95616; ^d^Department of Mathematics, University of California, Davis, CA 95616

**Keywords:** computational neuroscience, population coding, neural manifolds

## Abstract

Populations of neurons represent sensory, motor, and cognitive variables via patterns of activity distributed across the population. The size of the population used to encode a variable is typically much greater than the dimension of the variable itself, and thus, the corresponding neural population activity occupies lower-dimensional subsets of the full set of possible activity states. Given population activity data with such lower-dimensional structure, a fundamental question asks how close the low-dimensional data lie to a linear subspace. The linearity or nonlinearity of the low-dimensional structure reflects important computational features of the encoding, such as robustness and generalizability. Moreover, identifying such linear structure underlies common data analysis methods such as Principal Component Analysis (PCA). Here, we show that for data drawn from many common population codes the resulting point clouds and manifolds are exceedingly nonlinear, with the dimension of the best-fitting linear subspace growing at least exponentially with the true dimension of the data. Consequently, linear methods like PCA fail dramatically at identifying the true underlying structure, even in the limit of arbitrarily many data points and no noise.

## Significance

Information in the brain is collectively processed by very large populations of neurons. Finding shapes in neural population activity data has emerged as a powerful way to study how information is encoded and transformed by these large populations. Most common data analysis tools look for linear shapes in data, meaning lines, planes, and higher-dimensional flat analogues. We study two broad families of information-encoding strategies that are frequently observed in the brain. We show that the shapes corresponding to these information-encoding strategies are exceedingly nonlinear and thus would be completely missed by common data analysis methods.

Neural coding is distributed and redundant, with large populations of neurons collectively encoding relevant variables. Geometric frameworks provide a natural setting within which to formulate and test theories of population coding, along with tools that allow population structure to be extracted from data (1–10). In one particularly fruitful approach, data from a population of N neurons can be embedded in an N-dimensional space, with each axis corresponding to the activity of 1 neuron. The state of the population at each moment in time corresponds to a point in this N-dimensional space. Shared structure in the neural population code then corresponds to lower-dimensional shapes or, in the case of smooth responses, “manifolds” on which the data lie (11–19). Computation can be understood in terms of trajectories on these low-dimensional manifolds (20–28).

Given some population data with such lower-dimensional structure, a fundamental question asks how close the data lie to a low-dimensional linear subspace or hyperplane (i.e., is the lower-dimensional structure near-linear?). This question is of theoretical interest because the linearity or nonlinearity of the population data provides insight into the structure, robustness, and generalizability of the encoding (9, 11, 29). The linearity of data is also of great practical importance because methods that seek to fit a linear subspace to data, such as Principal Component Analysis (PCA) and Factor Analysis, are extremely widely used, whether to reveal structure in an unsupervised manner or as an initial data processing step before using regression and other supervised methods (1, 30).

Linear dimensionality reduction methods have a number of appealing features, including ease of interpretation, computational tractability, theoretical guarantees, and robustness. Moreover, linear methods are the foundation of a number of more advanced methods. For example, if a manifold is not well-fit by a linear subspace, a natural generalization seeks a set of linear subspaces that combine to describe the manifold (31, 32). On the other hand, using linear tools on highly nonlinear manifolds will be misleading. Thus, understanding when a manifold is linear or near-linear provides insight both into the coding strategy used by the corresponding brain region and determines the appropriate data analysis tools to be used.

In this study, we examine the linearity of the manifolds generated by common population codes. We show that the resulting manifolds are exceptionally nonlinear. For example, consider a population of neurons with Gaussian tuning to a stimulus with D features—each neuron shows maximum response at some preferred stimulus value and the response decreases as a Gaussian function of the distance between the current stimulus and the maximally preferred stimulus value. Since there are D independent dimensions of variation, the neural population responses at any moment in time can be represented by a D-dimensional vector and the data are contained within a D-dimensional manifold. We prove, however, that a linear subspace that contains 80% (or any other fixed fraction) of the variance in these data must have dimension that grows exponentially with D. This dimension can be in the many thousands even for small values of D. Thus, methods that seek to fit a linear subspace to data will greatly overestimate the dimension of the true manifold, even in the limit of arbitrarily many data points and neurons.

## Results

### Setup.

#### Low-dimensional population structure and neural manifolds.

**Fig. 1.**
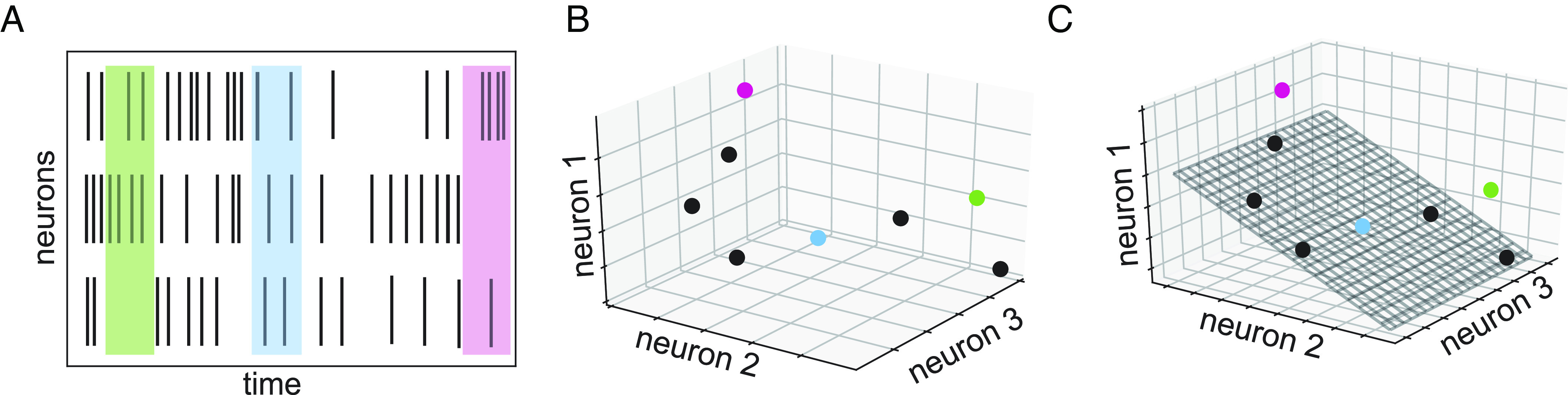
Schematic of low-dimensional structure in neural population data. (*A*) Spiking activity of 3 neurons over time. Shaded regions show three sample time bins, each used to compute an activity vector. (*B*) Activity represented as a collection of points in three-dimensional space. Colored points correspond to shaded regions in *A*. (*C*) Lower-dimensional linear structure in data, shown as a two-dimensional plane chosen to capture as much variance in the data as possible. Scatter of points (e.g., pink and green points) off of plane reflects variance that is not captured.

Given activity data from a population of N neurons over time, consider the population activity vector y(t), whose n-th entry $y_{n}\left(t\right)$ is the activity (e.g., number of spikes fired or fluorescence signal) of neuron n in a time window of size δt centered at time t. This activity vector can be seen as a point in an N-dimensional space, with each dimension corresponding to the activity of one of the recorded neurons (see schematic in Fig. 1). If the population activity is measured at T time points $t_{1},\cdots ,t_{T}$, then the recording yields T such activity vectors. The population activity vectors together form the N×T data matrix A, where $A_{\mathrm{ns}}$ is the activity of neuron n in time bin $t_{s}$.

The geometric picture corresponding to this collection of population activity vectors is a cloud of points in N-dimensional space, with each point corresponding to a moment in time (Fig. 1*B*). If the population of neurons shows structured activity, then the points will cluster around particular locations or trace out particular shapes. These shapes provide ways to explore and to reason about the nature of the underlying representation or computation.

In particular, assume that the population responses are driven by some time-varying D-dimensional latent variable x(t), meaning that y(t)=F(x(t))+ξ(t) for some function $F:R^{D}\to R^{N}$, and where ξ(t) is residual variance (e.g., noise). Here x(t) could be an external stimulus, attentional or arousal or satiety state, motor plan, decision variable, internal estimate of location, or any combination of such and other variables. In what follows, we will adopt the terminology of Jazayeri and Ostojic (9) and refer to this stimulus or other population variable as the “latent variable.”

Note that, ignoring noise, the location of any population activity vector in the N-dimensional space can be specified by at most D coordinates (i.e., the values of the latent variable x(t)), and thus, the data point cloud lies in a D-dimensional space. We will refer to D as the “intrinsic” dimension of the data, following previous work (9). Under some mild smoothness conditions on F and x, the data lie on a D-dimensional manifold, and we will thus refer to data “manifolds,” following common practice in the field (but the results do not require continuity and smoothness).

#### Populations with shared tuning curves.

**Fig. 2.**
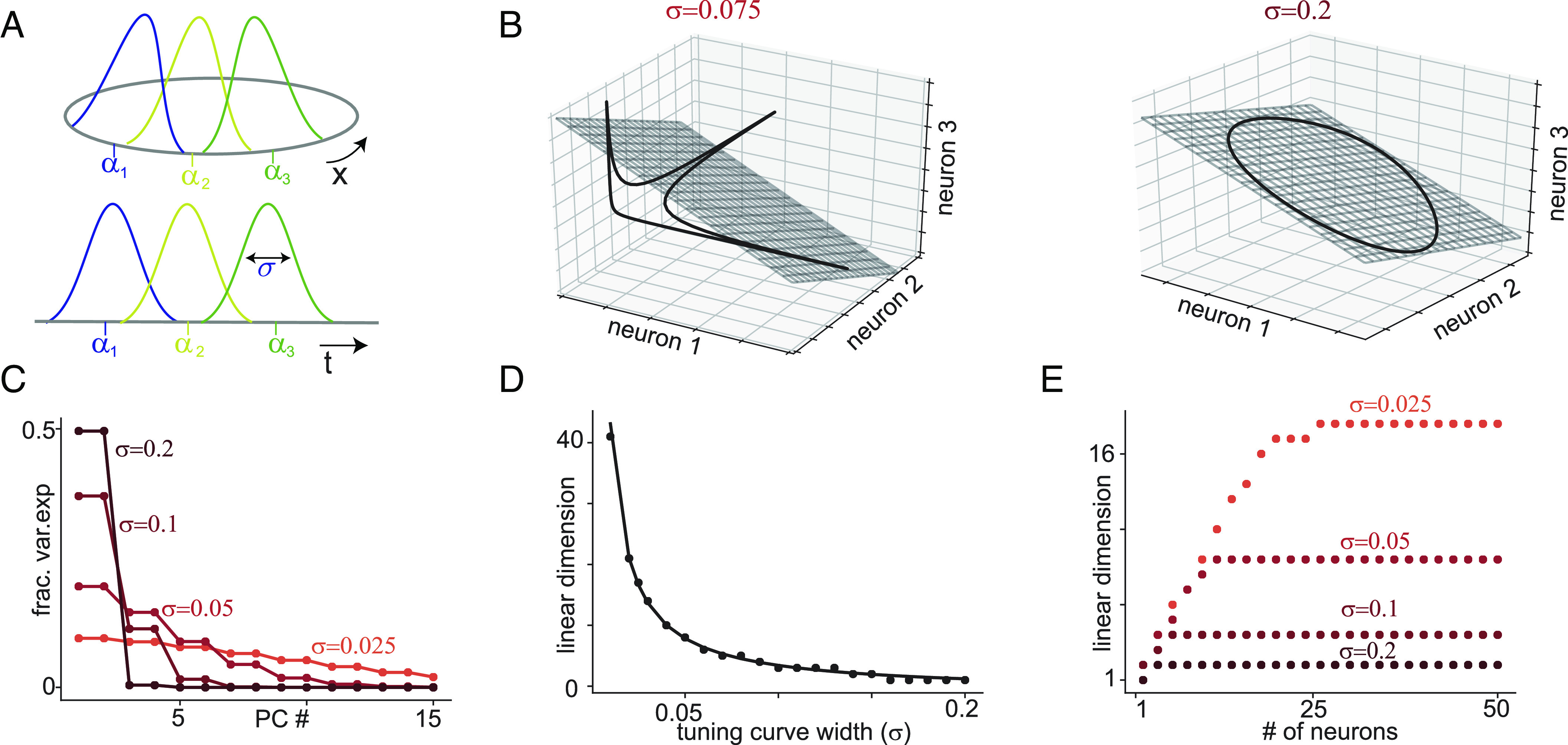
Translation-symmetric tuning to a one-dimensional variable and the inverse relationship between linear dimension and sparsity. (*A*) Gaussian tuning curves of 3 neurons encoding a circular (*Top*) or noncircular (*Bottom*) scalar stimulus variable. The noncircular variable example includes tuning to time, as in an epoch code. (*B*) Black line: Manifold formed by population activity of 3 neurons with Gaussian tuning to a 1-dimensional circular variable. Each axis shows the activity of 1 neuron. Gray: Best fitting 2D linear subspace (i.e., plane spanned by first two PCs). *Left* and *Right* show an example of narrow (σ=0.075) and broad (σ=0.2) tuning respectively. For (*C*)–(*E*), results shown are for Gaussian tuning to a circular variable, with uniformly spaced tuning curve centers. Circles show numerical simulations and lines show theoretical predictions. (*C*) Fraction of variance explained by each PC (equivalently, eigenvalues of covariance matrix) for a population of N=50 neurons. Different curves show different tuning curve widths. (*D*) Linear dimension of neural data against tuning curve widths, showing that linear dimension grows as 1/σ. (*E*) Linear dimension against number of neurons in a population for each tuning curve width, showing initial linear growth before saturation at the predicted values shown in *D*.

**Fig. 3.**
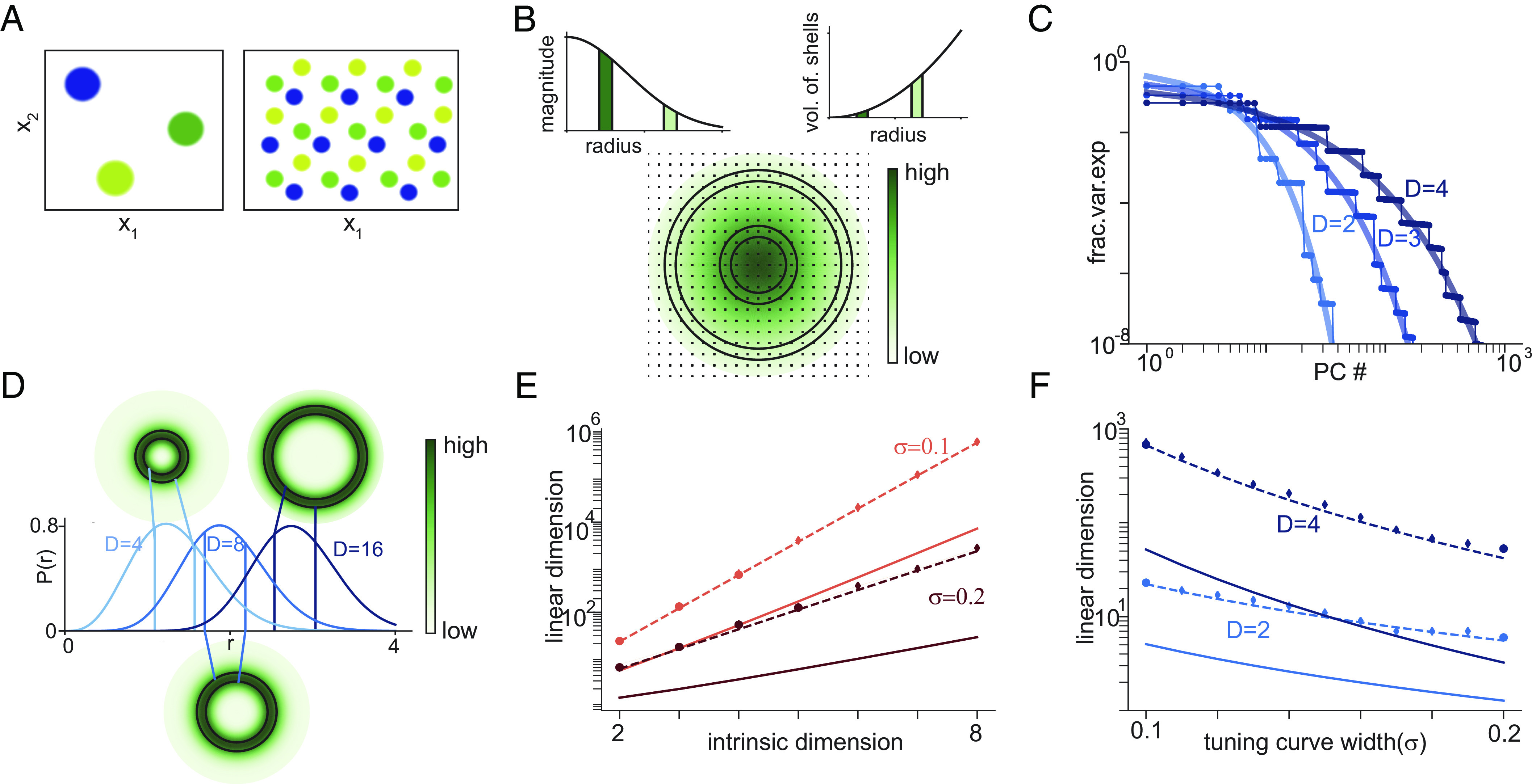
Translation-symmetric tuning to a multidimensional variable and exponential growth of linear dimension with intrinsic dimension. (*A*) Examples of 2D tuning curves, showing schematics of 3 different place cells with different tuning centers in a square arena (*Left*) and 3 grid cells with the same spacing but different phases (*Right*). (*B*) For Gaussian tuning curves, eigenvalues of the covariance matrix (variance along each PC) are values of a D-dimensional Gaussian at the lattice points of D-dimensional Fourier space. Each lattice point corresponds to one eigenvalue, and the colormap shows its value. *Left*: Decay of eigenvalues with distance from origin in Fourier space. *Right*: Number of eigenvalues contained in concentric shells of different radii. Circular shells on the plot highlight two sets of eigenvalues, with the corresponding magnitude and volume of shell shown as the shaded region in insets. For a shell close to the origin, the eigenvalues have a large magnitude but there are fewer eigenvalues as a consequence of the smaller volume. Away from the origin, the value of the eigenvalue is lower but there are more such eigenvalues. This tradeoff between eigenvalue magnitude and the number of eigenvalues of that magnitude explains the shape of the variance explained vs PC number curve. (*C*) Fraction of variance explained by each PC (or eigenvalues of covariance matrix) for D-dimensional Gaussian tuning curves and periodic boundary conditions along each dimension. Circles show numerical simulations, thin line represents prediction from Fourier transform of covariance matrix rows, and thicker lines represent theoretically predicted smooth interpolation. (*D*) Total probability mass at radius r for a D-dimensional Gaussian (i.e., density function of chi distribution), shown for three different values of D. Circular insets show concentric shells colored by total probability mass at that radius. The bulk of the probability mass lies in a shell of radius $∼\;\sqrt{D}/\sigma$. Thus, accounting for most of the variance requires considering all eigenvalues within a sphere of radius at least $∼\;\sqrt{D}/\sigma$. (*E*) Semilog plot of linear dimension (ϵ=0.05) vs. intrinsic dimension for Gaussian tuning curves with different widths. Circles show numerical results, solid lines show theoretical lower bound from median of chi distribution (applies whenever ϵ≤0.5), and dashed lines show semianalytic fit using chi distribution. (*F*) Semilog plot of linear dimension vs. tuning curve width. Circles and lines as in *E*.

**Fig. 4.**
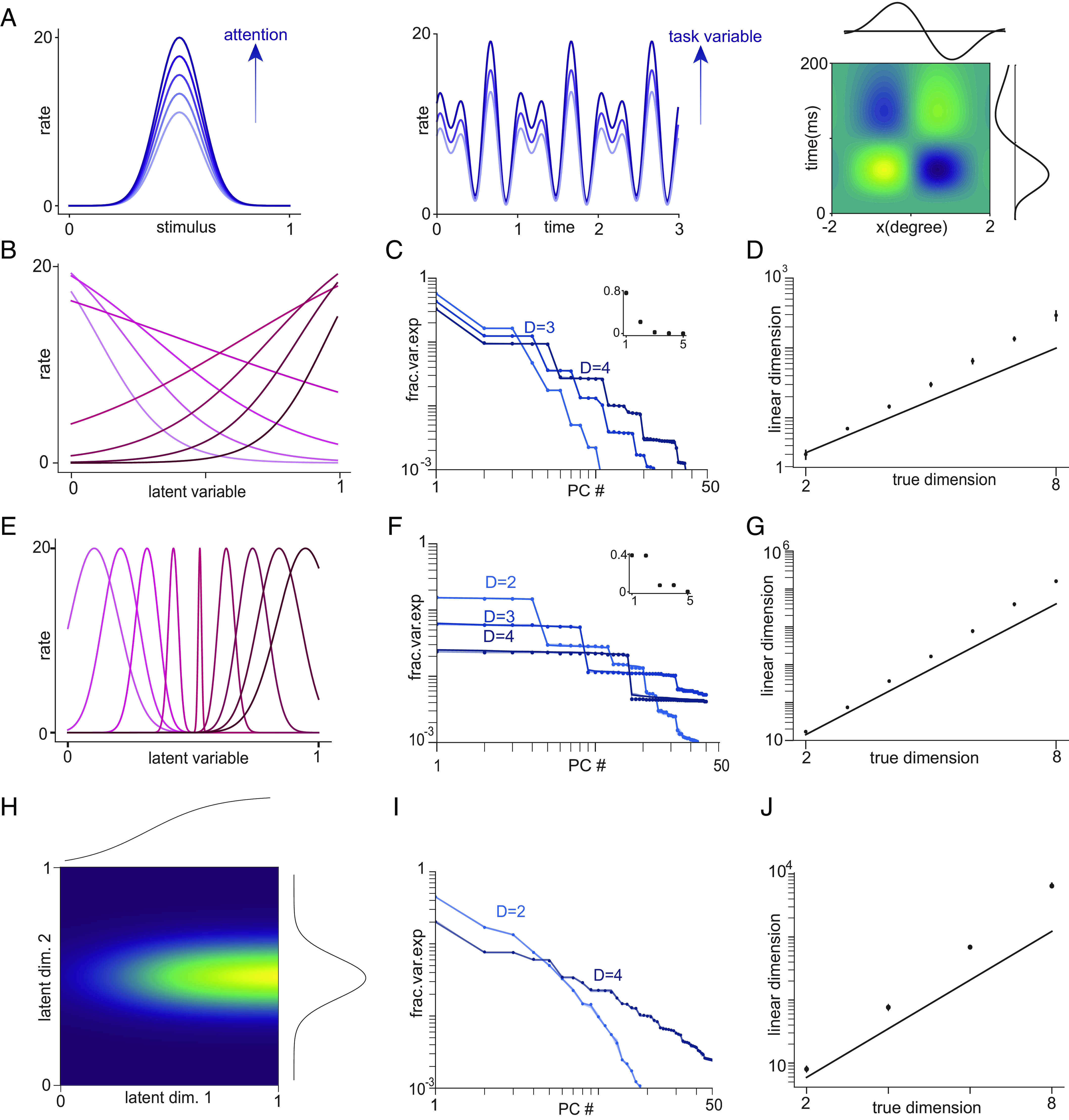
Multiplicative tuning and exponential growth of linear dimension with intrinsic dimension. (*A*) Schematics of common examples of multiplicative tuning. *Left*: Gain modulation of tuning to a sensory stimulus by attention. *Center*: Multiplicative modulation of epoch code by task variables. *Right*: Separable spatiotemporal receptive field of retinal ganglion cell as product of spatial (horizontal) and temporal tuning (vertical). Panels (*B*)–(*D*) show results from a multiplicative tuning model where tuning along each dimension is sigmoidal. (*B*) Sample tuning along each dimension. Tuning curves are sigmoidal with slopes chosen uniformly in range [−5,5] and centers evenly distributed in [0,1]. (*C*) Fraction of variance explained vs. PC number for the model shown in *B* for different values of intrinsic dimension (D). Circles show numerical simulations and lines show the result from the tensor product of 1D tuning curves. The inset shows the eigenvalues in the 1D case. (*D*) Linear dimension against intrinsic dimension for the data in *C*. Circles show simulations, and the solid line shows theoretical lower bound of $2^{D(H-0.05)}$, where H is the entropy of the eigenvalue distribution shown in the *Inset* of panel (*C*). Panels (*E*)–(*G*) show results from a multiplicative tuning model where tuning along each dimension is Gaussian. The Gaussians are not translation-symmetric and the width of the Gaussian depends on position, with tuning sharpest at the center of the stimulus space (as in visual receptive fields). (*E*) Sample tuning along each dimension. (*F*), (*G*) As in (*C*), (*D*) but for the model shown in *E*. Panels (*H*)–(*J*) show results from a multiplicative tuning model where factors are an equal mixture of sigmoidal and Gaussian (i.e., a hybrid of the models shown on the previous 2 rows). (*H*) Sample tuning curve of one neuron tuned to a 2D latent variable, with sigmoidal tuning along one dimension (horizontal) and Gaussian tuning along the other (vertical). (*I*), (*J*) As in (*C*), (*D*) but for the model shown in (*H*).

In the setting above, the response of the nth neuron is determined by $F_{n}$, the nth component of F. $F_{n}$ thus captures the tuning of the nth neuron to the latent variable x. In many neural populations, these tuning curves take a similar shape or functional form across neurons but differ in their preferred stimulus, width of selectivity, or other parameters (examples in Figs. 2*A*, 3*A*, and 4*A*). Such shared tuning curve structure is common in topographically organized sensory regions (33–38) and in populations that show spatial tuning, such as place, grid, and head direction cells (39–41); it has also been found for more abstract quantities, such as among neurons tuned to numbers (42, 43) and to decision variables such as accumulated evidence (18, 44).

If tuning curves take a similar functional form, the activity of the nth neuron at time t can be modeled by $y_{n}\left(t\right)=f\left(x\left(t\right),\alpha_{n}\right)$, with n=1,⋯,N. Here f is a tuning curve function representing the shared shape of the tuning curve, $\alpha_{n}$ is a vector of tuning curve parameters (e.g., preferred stimulus or tuning curve center of a sensory neuron, phase and period of a grid cell, preferred value of a decision variable, etc.), and, as before, x is the time-varying D-dimensional latent variable that underlies the population responses. Note that while such tuning curves were historically applied to describe the relationship between neural activity and an external variable, the latent variable can also be an internal cognitive variable (18, 44–49) or even an abstract statistical construct (13, 16, 50) that captures network interactions. For example, in a ring attractor network that encodes heading direction, the latent variable is the network’s estimate of heading direction, and the tuning of each neuron emerges from recurrent network interactions (40, 51, 52). Thus the model is quite flexible and captures a variety of population codes.

#### Linearity of point clouds and manifolds.

The most natural structure to seek in population data is linearity, corresponding to finding a lower-dimensional subspace (hyperplane) that contains the data (see schematic in Fig. 1*C*). If the dimension of this subspace is L, linear structure corresponds to finding L vectors $v_{1},\cdots ,v_{L}$ whose weighted sums account for the data. That is, any data point $y\left(t\right)=\sum_{l=1}^{L}a_{l}\left(t\right)v_{l}$, where $a_{l}\left(t\right)$ is the time-varying contribution of the l-th vector. Equivalently, the rank of the data matrix A is L.

In the presence of noise, data points will not lie exactly in a lower-dimensional linear subspace. Even in the absence of noise, a set of data points may not lie exactly in a linear subspace but might be close enough to be approximated by a linear subspace for practical purposes. Thus, it is typical to look for a linear subspace that captures most of the spread in the data while allowing for some scatter in the data around the subspace (Fig. 1*C*). Equivalently, one looks for L basis patterns that can approximately sum to any population activity vector $\left(y\left(t\right)\approx \sum_{l=1}^{L}a_{l}v_{l}\left(t\right)\right)$ or for a rank L matrix $A_{L}$ such that $||A-A_{L}\left|\right|$ is small in some appropriate norm (usually 2-norm or Frobenius norm).

More precisely, we define the (1−ϵ)-linear dimension $L_{1-\epsilon }$ of a matrix A to be the smallest R such that there exists a rank R matrix $A_{R}$ for which $||A-A_{R}{\left|\right|}_{F}^{2}{<\epsilon ||A||}_{F}^{2}$ (this quantity is related to the ϵ-rank of A (53)).

This definition of linear dimension corresponds to common practice in neural data analysis, where it is typical to perform PCA and estimate the dimension of data as the number of principal components (PCs) required to explain some high fraction (i.e., 1−ϵ in our notation) of the variance (1, 9, 54). Thus, for example, what we call the 0.8-linear dimension is the number of PCs required to account for 80% of the variance in the data.

The best rank R approximation to the data matrix A is $\sum_{k=1}^{R}\sigma_{k}u_{k}v_{k}^{T}$, where $\sigma_{k}$ is the k-th singular value of A, and $u_{k}$, $v_{k}$ are the kth left and right singular vectors respectively. The remaining variance $||A-A_{R}{\left|\right|}_{F}^{2}=\sum_{k=R+1}^{N}\sigma_{k}^{2}$. In other words, the matrix A has (1−ϵ)-linear-dimension $L_{1-\epsilon }$ if

[1] $$\begin{array}{c} \sum_{k=1}^{L_{\epsilon }}\sigma_{k}^{2}/\sum_{k=1}^{N}\sigma_{k}^{2}\geq 1-\epsilon \;\;\text{but}\;\sum_{k=1}^{L_{\epsilon }-1}\sigma_{k}^{2}/\sum_{k=1}^{N}\sigma_{k}^{2}<1-\epsilon . \end{array}$$

The singular values of A can also be calculated from the eigenvalues of the (non-mean-subtracted) covariance matrix $AA^{T}$, which is the matrix of covariances between neurons averaged over time, or $A^{T}A$, which is the matrix of covariances between data points averaged over neurons. The k-th eigenvalue of each of these matrices is $\lambda_{k}=\sigma_{k}^{2}$ (for k≤N, assuming more time points than neurons).

Constructing such a low rank approximation to the data matrix (or, equivalently, fitting a linear subspace to the data point cloud) is the foundation of commonly used dimensionality reduction methods such as PCA and Factor Analysis. Moreover, a number of nonlinear dimensionality reduction techniques rely on approximating the data point cloud or manifold by a family of linear subspaces (31, 32, 55). Such methods will be expected to perform well when the data point cloud or manifold is near-linear and poorly when the data manifold is highly nonlinear.

#### Overview of approach.

In this study, we consider a population of N neurons whose activity is driven by a D-dimensional real-valued latent variable x, with firing rates given by tuning curve functions $f(x,\alpha_{n})$. Thus, the intrinsic dimension of neural activity is D. For several choices of tuning curve function we lower bound the (1−ϵ)-linear dimension of the activity (equivalently the number of PCs required to explain a (1−ϵ) fraction of the data variance) and show that it is very large, growing at least exponentially with D.

We assume that x takes on all possible values in a compact subset of $R^{D}$ and that f is continuous and one-to-one, so that in the absence of noise population responses lie on a D-dimensional manifold. However, the approach can be naturally generalized to finding a linear subspace that contains point cloud data instead, and thus extends to cases like noncontinuous values of the latent variable.

We consider firing rates and ignore noise so that the response of the nth neuron is given exactly by the mean firing rate, $y_{n}\left(t\right)=f\left(x,\alpha_{n}\right)$, and thus, the time window around t in which the rate is measured does not affect the results (as long as it is small on the timescale at which x changes). In the absence of noise, PCA and Factor Analysis are equivalent and our results thus apply to both methods (as well as to methods like Probabilistic PCA). Given that our results lower bound the linear dimension, including noise would simply strengthen our results by making neural activity more high-dimensional. Thus, results reflect fundamental lower bounds on the dimensionality of neural activity rather than a lack of data and would not change if neural responses were averaged over multiple stimulus presentations.

We consider tuning curve functions $f(x,\alpha_{n})$ with certain symmetries and use these symmetries to exactly or approximately calculate the eigenvalues of the neuron-neuron covariance matrix (which are also the squared singular values of the data matrix and, when normalized, the fractions of variance explained by the different PCs). We then count the number of eigenvalues needed to account for a (1−ϵ) fraction of the variance in activity, for some small ϵ. Our results are not sensitive to the choice of ϵ and in general apply when ϵ<0.5.

To define the covariance matrix and calculate its eigenvalues, it will be convenient to first define the correlation profile function c between neurons with tuning parameters $\alpha_{m}$ and $\alpha_{n}$ to be $c\left(\alpha_{m},\alpha_{n}\right)=E_{x}\left[f\left(x\left(t\right),\alpha_{m}\right)f\left(x\left(t\right),\alpha_{n}\right)\right]$, where the expectation is taken over the values of the latent variable x. This is simply the (non-mean-subtracted) covariance between the neurons. For a population of N neurons, the N×N covariance matrix, C, has (m,n)th entry $C_{\mathrm{mn}}=c\left(\alpha_{m},\alpha_{n}\right)$.

Note that we primarily consider the non-mean-subtracted covariance matrix. Methods such as PCA often first subtract the mean from data. In *SI Appendix*, section 1 and Fig. S2 we show that if $L_{1-\epsilon }$ is the linear dimension of the non-mean-subtracted data, then $L_{1-\epsilon }-1$ is a lower bound on the linear dimension of the mean-subtracted data (this is a consequence of the Weyl inequalities relating the eigenvalues of perturbed matrices). Thus, our lower bounds on linear dimension apply to mean-subtracted data, and in particular scaling arguments hold.

### Translation-Symmetric Population Codes.

In many brain regions, responses to the latent variable are given by tuning curves whose shape is approximately the same across neurons but with the tuning curve shifted or centered around a different region of latent variable space for each neuron (33–41). Examples of approximate translation-symmetric tuning range from early sensory systems, such as orientation tuning in area V1 (35, 56), to cognitive systems, such as spatial tuning in hippocampal place cells and entorhinal grid cells (39, 41, 45, 46), epoch codes and hippocampal time cells (57), and tuning to abstract variables such as number (42, 43).

In this setting, the response of neuron n at time t is determined by the difference between the current value of the latent variable, x(t), and the neuron’s preferred value $\alpha_{n}$ (note that for convenience we refer to the “center” or “preferred stimulus” of the tuning curve but the parameter $\alpha_{n}$ more generally can simply index the shift of the tuning curve from an arbitrarily chosen reference tuning curve function as, for example, with the phase of a grid cell). If $x\in R^{D}$ is the latent D-dimensional variable, then the tuning curve parameter α is also in $R^{D}$, and the tuning curve function f(x,α)=g(x−α) for some function g [equivalently, given some $\delta \in R^{D}$, f(x,α)=f(x+δ,α+δ)]. Different neurons have different preferred values, together tiling the space of possible values.

#### One-dimensional translation-symmetric populations and the sparsity-linear dimension uncertainty relation.

First, consider a translation-symmetric population of N neurons where the encoded variable x and the tuning parameter for each neuron $\alpha_{n}$ are drawn from a one-dimensional (1D) circular space (i.e., $S^{1}$), with points in the space parameterized by [0,1), Fig. 2*A*, *Top*. For example, x could be the angle of orientation of a bar, the direction of motion of a stimulus, or the head direction of a moving animal; correspondingly, $\alpha_{n}$ could be the center of the tuning curve of the nth neuron. If x evenly samples the space, then the correlation profile c for two neurons depends only on the difference $\alpha_{m}-\alpha_{n}$ between tuning parameters. We slightly overload notation and write the correlation profile as $c\left(\alpha_{m},\alpha_{n}\right)=c\left(\delta \right)$ where $\delta =\alpha_{m}-\alpha_{n}$ and the function c is periodic with period 1.

If the tuning parameters evenly tile the space, then the entries of the covariance matrix are

[2] $$\begin{array}{c} C_{\mathrm{mn}}=c\left(\alpha_{m}-\alpha_{n}\right)=c\left(\left(m-n\right)/N\right). \end{array}$$

The matrix C is circulant, meaning that each row is a shifted copy of the row above. It is well known (and easy to show, see *SI Appendix*, section 2.1) that the eigenvalues of C are given by the Fourier transform of c, the function used to generate each row (4, 29, 58–60). Thus, the pth eigenvalue is

[3] $$\begin{array}{c} \lambda_{p}=\sum_{l=0}^{N-1}c\left(l/N\right)e^{-2\pi ipl/N}\approx N\int_{0}^{1}c\left(x\right)e^{-2\pi ipx}dx, \end{array}$$

where $i=\sqrt{-1}$ and the approximate equality improves as N gets larger^*^.

If tuning curve centers are not evenly spaced, the corresponding matrix is no longer circulant, but the eigenvalues are still approximately given by the Fourier transform of c. If tuning curve centers are randomly sampled, this approximation converges rapidly as the number of neurons increases. Similarly, consider a stimulus space that is 1D but not circular, Fig. 2*A*, *Bottom*. A natural example of this setting is tuning to time, as in an epoch code or the responses in a synfire chain. The corresponding matrix is Toeplitz and the same eigenvalue relationship approximately holds (58, 61–64). Thus, quite generally, the profile of eigenvalues is given by the Fourier transform of the correlation profile (see *SI Appendix*, Fig. S3 for numerical confirmation of the approximate relationships).

As a specific example, consider translation-symmetric Gaussian tuning, which is a common approximate model for tuning curves across multiple systems like orientation selective neurons in visual cortex V1 (65) and place cells in the hippocampus (39). Note that Gaussian tuning is technically defined on an infinite interval and thus for periodic boundary conditions is only a good approximation if tuning curves are not too wide, so that the periodicity can be ignored (but Gaussians could be replaced by von Mises functions to model wider tuning).

For Gaussian tuning the nth neuron’s response is

[4] $$\begin{array}{c} y_{n}\left(t\right)=K_{1}\exp\left(-\frac{{\left(x\left(t\right)-\alpha_{n}\right)}^{2}}{2\sigma^{2}}\right), \end{array}$$

where $K_{1}$ is the maximum firing rate and σ is the width of the tuning curve. The corresponding manifolds for a population of 3 neurons are shown in Fig. 2*B*. The covariance between the mth and nth neurons is $C_{\mathrm{mn}}=K_{2}\exp\left(-\frac{\delta_{\mathrm{mn}}^{2}}{4\sigma^{2}}\right)$. Here $K_{2}$ is a constant and $\delta_{\mathrm{mn}}$ is the difference between the tuning curve centers (calculated accounting for the circular boundary conditions). The eigenvalues of the covariance matrix for large N are given by

[5] $$\begin{array}{cc} \lambda_{p} & =K_{3}e^{-4\pi^{2}\sigma^{2}p^{2}}, \end{array}$$

where $K_{3}$ is a constant, as shown in Fig. 2*C* (see *SI Appendix*, sections 2.3 and 2.4 for the calculation). Thus, the eigenvalue profile is Gaussian with variance inversely proportional to the width of the tuning curve.

The (1−ϵ)-linear dimension is the smallest $L_{1-\epsilon }$ such that $\sum_{p=0}^{L_{1-\epsilon }}\lambda_{p}\geq \left(1-\epsilon \right)\sum_{p=0}^{N}\lambda_{p}$. If N is not too small, these sums can be approximated by Gaussian integrals yielding the condition

[6] $$\begin{array}{c} L_{1-\epsilon }=\frac{1}{\sigma }\frac{{\;\mathrm{erf}\;}^{-1}\left(1-\epsilon \right)}{\pi }. \end{array}$$

Thus, to explain a constant fraction of the variance, the linear dimension generically grows as 1/σ, where σ is the tuning curve width, as shown in Fig. 2*D*. In particular, $L_{0.95}$ corresponds to a 95% CI for a Gaussian distribution and is thus $\frac{1.96}{\sqrt{2}\;\pi \sigma }$. Note that in practice, if N (the number of sampled neurons) is small then linear dimension will be bounded by N but will increase toward the true linear dimension as N increases, as shown in Fig. 2*E*.

More generally, so-called uncertainty principles relate the spread, sparsity, entropy, or concentration of a function to that of its Fourier transform (66–69). These principles imply that if the tuning curves (and hence the correlation profile) are sparse, fall off rapidly around their preferred values, or are concentrated on relatively small subsets of latent variable space, then λ will decay slowly with increasing p and have many significant nonzero entries. If λ decays slowly, then the number of eigenvalues needed to capture most of the variance will be high. Consequently, the linear dimension will be large and the manifold will be highly nonlinear.

Gaussian functions are lower bounds for several uncertainty principles, and thus, the 1/σ scaling will be a lower bound across a wide class of tuning curve shapes, in particular those with firing localized to some region of latent space (or the manifold). However, translation-symmetry and the uncertainty principles do not require tuning curves to be unimodal or localized, and highly nonlinear manifolds are expected whenever the tuning curves (and hence the covariance profile) are concentrated on comparatively small subsets even if these tuning curves are not localized to a single interval or region.

We highlight two useful uncertainty principles that apply in this more general setting. First, if the covariance profile c has K nonzero entries (i.e., is K-sparse), then the eigenvalue profile has at least N/K nonzero entries (67). Consequently, if K is small then there are many nonzero eigenvalues. Second, if a fraction $1-\hat{\epsilon }$ of the covariance profile is concentrated on a set S of size K (meaning that $\sum_{\delta \in S}|c\left(\delta \right)|>\left(1-\hat{\epsilon }\right)\sum_{\delta \in \Omega }\left|c\left(\delta \right)\right|$, where Ω is the domain of the covariance profile), then the smallest set that contains 1−ϵ of the eigenvalue mass has size at least $N\left(1-\hat{\epsilon }\right)\left(1-\epsilon \right)/K$ (67, 69). The size of this set is just the (1−ϵ)-linear dimension and consequently linear dimension again grows inversely with K. Sparse coding thus generically implies high linear dimension.

In practice, for systems with relatively broad tuning curves and for which the latent variable is low-dimensional, such as head direction cells (52, 70) or ventral hippocampal place cells (71, 72), the overestimate of intrinsic dimension by linear dimension may not be too large. However, in many systems, both sensory and cognitive, neurons respond to a small fraction of possible values of the latent variable. For example, foveal V1 cells in the primate cover less than a degree of visual space (35), and rodent hippocampal place cells can cover under 1% of the area of large environments (72, 73). Similarly, cells that are tuned to complex visual stimuli such as faces or other objects tend to show sparse responses (74, 75), thus covering only a small portion of stimulus space. In these settings, the manifold is likely to be highly nonlinear and linear dimensionality will greatly overestimate intrinsic dimensionality.

#### Multidimensional translation-symmetric tuning and exponential growth of linear dimension.

As with the one-dimensional case, when a higher-dimensional variable x is encoded with translation-symmetric tuning curves (schematic in Fig. 3*A*), the covariance profile is also translation-symmetric and the eigenvalues of the covariance matrix are given by the Fourier transform of the covariance profile. Consequently, as in the 1D case, tuning curves that are sharper or concentrated on smaller sets will yield more slowly decaying eigenvalue profiles and hence higher linear dimension. However, the linear dimension will depend strongly on D, the intrinsic dimension of the latent variable. We first examine this interaction in the Gaussian case, before drawing general conclusions.

Consider a population of N neurons with translation-symmetric Gaussian tuning to an underlying D-dimensional latent variable x that takes values within [0,1] along each dimension. The tuning curve for the nth neuron is centered at $\alpha_{n}$. By an appropriate choice of basis for x, the covariance matrix of the Gaussian tuning curve can be assumed diagonal. For simplicity, we assume circular boundary conditions and that x is scaled so that tuning curves have equal width σ in all directions. Thus, the response of the n-th neuron is

[7] $$\begin{array}{cc} y_{n}\left(t\right) & =K_{1}\exp\left(-\frac{||x(t)-\alpha_{n}{\left|\right|}^{2}}{2\sigma^{2}}\right), \end{array}$$

where $K_{1}$ is the maximum firing rate. The corresponding correlation profile is also Gaussian. If tuning curve centers are equally spaced, then the covariance matrix has (m,n)th entry $C_{\mathrm{mn}}=K_{2}\exp\left(-\frac{{\left||\delta_{\mathrm{mn}}|\right|}^{2}}{4\sigma^{2}}\right)$, where $K_{2}$ is a constant and $\delta_{\mathrm{mn}}$ is the difference between the tuning curve centers $\alpha_{m}$ and $\alpha_{n}$ (accounting for the circular boundary conditions). For large N, the eigenvalues are given by (*SI Appendix*, sections 2.3 and 2.5)

[8] $$\begin{array}{c} \lambda_{p}=K_{3}\exp\left(-4\pi^{2}\sum_{d=1}^{D}p_{d}^{2}\sigma^{2}\right)=K_{3}\exp(-4\pi^{2}\sigma^{2}{\left|p\right|}^{2}). \end{array}$$

Here $K_{3}$ is a constant. The eigenvalues are indexed by a D-dimensional vector p with dth entry $p_{d}\in \left[-N_{d}/2,\cdots ,N_{d}/2\right]$, where $N_{d}$ is the number of tuning curve centers along dimension d (assumed the same for simplicity). Note that these eigenvalues are given by a multivariate Gaussian evaluated at the integer lattice points of a D-dimensional rectangle with side lengths $N_{d}$, Fig. 3*B*.

The magnitude of an eigenvalue depends only on the magnitude of p, and thus, the eigenvalues can be ordered by smallest to largest distance from the origin in p-space. There will be multiple eigenvalues with the same magnitude, corresponding to the same value of |p|. The number of eigenvalues of a given magnitude will increase with distance from the origin. Thus, moving away from the origin, there will be more eigenvalues but of smaller magnitude (schematic in Fig. 3*B*).

When ordered by their magnitude, the eigenvalue profile thus shows a step-like shape, Fig. 3*C*. A smoothly interpolating function for the eigenvalue profile can be derived by noting that the eigenvalue profile is spherically symmetric in p-space, and thus, the number of eigenvalues of a given magnitude depends on the number of lattice points at the corresponding radius. Interpolating this number by the volume of a D-dimensional ball yields the interpolating function

[9] $$\begin{array}{c} \lambda_{p}∼\exp\left(-\frac{2\sigma^{2}{\left(\pi D^{(D+1)/2}p\right)}^{2/D}}{e}\right), \end{array}$$

where the scalar p now indexes the eigenvalues from 1 to N (the total number of neurons) (see *SI Appendix*, section 2.5 and also see ref. 4 for a similar argument). The eigenvalues decay first slowly and then rapidly, showing a transition between a power-law-like and an exponential regime.

To convert these eigenvalue profiles into linear dimension, again note that the covariance profile is radially symmetric. Thus, we first seek the smallest radius r such that a fraction (1−ϵ) of the total eigenvalue mass lies in a sphere of radius r, and then count the number of eigenvalues in that sphere. The scaling of this radius with D can be derived by observing that the probability mass of a D-dimensional Gaussian concentrates in a shell of radius $\sqrt{D}/\sigma$ around the origin, as shown in Fig. 3*D* (we provide further details and calculate the radius more exactly using a χ-distribution in *SI Appendix*, section 2.5)

Thus, any sphere that captures a significant fraction of the probability mass must grow as $r∼\sqrt{D}/\sigma$.^†^ The number of eigenvalues (or lattice points) in a D-dimensional sphere of radius r grows as the volume, approximately as $\frac{1}{\sqrt{D\pi }}{\left(\frac{2\pi e}{D}\right)}^{D/2}r^{D}$. Consequently, as shown in Fig. 3*E* and *F*, the linear dimension grows as $\frac{1}{\sqrt{D\pi }}{\left(\frac{K_{4}}{\sigma }\right)}^{D}$, where $K_{4}$ is a constant. The solid lines in Fig. 3*E* and *F* show an exponentially growing theoretical lower bound that is derived from the median of a chi distribution and is valid whenever ϵ<0.5 (i.e., capturing at least 50% of variance).

As long as tuning curves are not too wide, this scaling of linear dimension is extremely rapid, growing exponentially with intrinsic dimension. Moreover, for exponential scaling to break down tuning curves must be very wide—enough that any individual neuron responds significantly to all possible values of the latent variable (*SI Appendix*, sections 2.5 and 4 and Fig. S4). Thus, exponential scaling will be the default and even relatively low-dimensional Gaussian manifolds with broad tuning will have very high linear dimension. For example, as shown in Fig. 3*E*, a population of neurons with broad Gaussian tuning to an 8 dimensional latent variable has linear dimension $L_{0.95}>6\times 10^{5}$.

The structure of the argument presented above is quite general, relying on the interaction between the decay of the eigenvalue magnitude with distance from the origin and the growth of volume with radius—while eigenvalues decay rapidly with distance, the growth of volume means that the radius of a sphere that captures some significant fraction of the total mass of eigenvalues must grow with D. The argument thus extends to other sparse tuning curves even if non-Gaussian.

Exponential or faster scaling for sparse tuning curves can be more generally derived from uncertainty principles. Analogous to the 1D setting, the results of ref. 69 can be used to show that if a fraction $1-\hat{\epsilon }$ of the covariance profile is concentrated on a set S of size K, then the smallest set that contains 1−ϵ of the eigenvalue mass (i.e., the (1−ϵ)-linear dimension) has size at least $N_{D}^{D}\left(1-\hat{\epsilon }\right)\left(1-\epsilon \right)/K$ (where as before $N_{D}$ is the number of tuning curve centers per dimension, assumed the same for simplicity). For the case of Gaussian tuning, the size of the set containing 50% of the covariance profile can be upper bounded by the number of points in a sphere of radius $\sigma \sqrt{D}$, and this when combined with the uncertainty principle again yields exponential scaling.

In the more realistic case where tuning curves are truly localized, meaning that each neuron’s tuning curve decays to zero within a finite length (rather than, e.g., the small but infinite tails of a Gaussian function), most of the mass of the covariance profile is contained within a sphere of fixed radius, independent of dimension. In this setting, the linear dimension grows as $L_{1-\epsilon }\approx {\sqrt{D}}^{D}\left(1-\epsilon \right)$ (*SI Appendix*, section 2.6), and thus grows supraexponentially with dimension.

### Multiplicative Tuning.

We next consider tuning curve models where tuning to the latent variable can be written as a product over 1D or lower-dimensional factors. That is, the tuning curve function is of the form

[10] $$\begin{array}{c} y_{n}\left(t\right)=f\left(x\left(t\right),\alpha_{n}\right)=\prod_{d=1}^{D}f_{d}\left(x^{d}\left(t\right),\alpha_{n}^{d}\right). \end{array}$$

Here $x^{d}$ and $\alpha_{n}^{d}$ are the d-th components of the vectors x and $\alpha_{n}$, and the $f_{d}$’s are scalar functions. For simplicity, we assume that each factor $f_{d}$ is a function of a scalar variable $x^{d}$. However in general the $f_{d}$’s could be multivariate functions of disjoint sets of multiple variables and similar results hold.

As an example of such tuning, common models of attention involve multiplicative gain modulation (76). Thus the latent variable includes both the current stimulus value and the value of the attentional signal, and the response of a neuron can be written as a product of stimulus tuning and the response to the attentional signal (Fig. 4*A*, *Left*). Another example is the multiplicative modulation of an epoch code by task variables observed in some decision-making tasks (Fig. 4*A*, *Center*) (77, 78). For a third example, the spatiotemporal receptive fields of early visual cells are often decomposed as a product of the spatial part and the temporal part (Fig. 4*A*, *Right*) (79). More generally, multidimensional tuning curves that are not multiplicative may be able to be approximated by a product of lower-dimensional factors in a so-called “mean field” or separable approximation.

Let m and n be two neurons with parameter vectors $\alpha_{m}$ and $\alpha_{n}$. If the sampling of the latent variable is independent across dimensions and boundary conditions are rectangular, then the covariance between these neurons can be written as

[11] $$\begin{array}{cc} c(\alpha_{m},\alpha_{n}) & =\int dx\;p(x)f(x,\alpha_{m})f(x,\alpha_{n})\\ & =\prod_{d=1}^{D}\int dx^{d}\;p\left(x^{d}\right)f_{d}\left(x^{d},\alpha_{m}^{d}\right)f_{d}\left(x^{d},\alpha_{n}^{d}\right)\\ & =\prod_{d=1}^{D}c^{d}\left(\alpha_{m}^{d},\alpha_{n}^{d}\right), \end{array}$$

where $p\left(x\right)=\prod_{d}p\left(x^{d}\right)$ is the distribution of latent variable values, $\alpha_{m}^{d}$ and $\alpha_{d}^{n}$ are the dth components of the parameter vectors $\alpha_{m}$ and $\alpha_{n}$, and we have defined $c^{d}\left(\alpha_{m}^{d},\alpha_{n}^{d}\right)=\int dx^{d}p\left(x^{d}\right)f_{d}\left(x^{d},\alpha_{m}^{d}\right)f_{d}\left(x^{d},\alpha_{n}^{d}\right)$. While the function c yields the covariance between any two neurons, each function $c^{d}$ yields the portion of the covariance that comes from the similarity of responses along the dth dimension.

As in the translation-symmetric case, we assume that the tuning curve parameters tile the space, forming the points of a lattice with $N_{d}$ tuning curve parameters along the dth dimension. Let the parameters along the d-th dimension be $\left\{\beta_{1}^{d},\cdots ,\beta_{N_{d}}^{d}\right\}$. Note that these parameters do not need to be equally spaced and that $N_{d}$ and the specific choice of parameters can differ across dimensions. The dth component of any tuning curve parameter vector (e.g., $\alpha_{m}^{d}$) is drawn from $\left\{\beta_{1}^{d},\cdots ,\beta_{N_{d}}^{d}\right\}$, and as a population, the parameter vectors span all $N=\prod_{d}N_{d}$ combinations of parameters.

If the tuning curve parameters tile the space in this way, the N×N covariance matrix of the data can be written in terms of a set of smaller matrices, $C^{d}$ that capture the component of covariance along each dimension. Here, each matrix $C^{d}$ is $N_{d}\times N_{d}$ and has entries $C_{\mathrm{rs}}^{d}=c^{d}\left(\beta_{r}^{d},\beta_{s}^{d}\right)$. We then have $C={⊗}_{d=1}^{D}C^{d}$, where ⊗ indicates the tensor product (see *SI Appendix*, sections 3.1 and 3.2 for more details).

Now let $\left\{\gamma_{1}^{d},\gamma_{2}^{d},\cdots ,\gamma_{N_{d}}^{d}\right\}$ be the eigenvalues of $C^{d}$. The eigenvalues of C are all possible products of one eigenvalue from each $C^{d}$ and so take the form $\prod_{d}\gamma_{p_{d}}^{d}$, where $\gamma_{p_{d}}^{d}$ is the $p_{d}$th eigenvalue of $C^{d}$ and each $p_{d}$ ranges over 1 to $N_{d}$.

#### The linear dimension of multiplicative models grows exponentially with intrinsic dimension.

Note that the eigenvalues of each component $C^{d}$ (when appropriately normalized) can be interpreted as the outcome probabilities of a categorical random variable $Z_{d}$, taking values in $\left\{1,\cdots ,N_{d}\right\}$ with $P\left(Z_{d}=k\right)=\gamma_{k}^{d}$. Moreover, the eigenvalues of the covariance matrix itself can be interpreted as the outcome probabilities of the joint random variable $Z=(Z_{1},\cdots ,Z_{D})$. For simplicity, here, we present the case where the tuning along each dimension has the same functional form. Thus each component $C^{d}$ has the same eigenvalues, which we denote $\left\{\gamma_{1},\gamma_{2},...,\gamma_{N_{D}}\right\}$, and each $Z_{d}$ has the same distribution. However, the argument extends to the case when tuning to different dimensions takes different shapes, as shown in *SI Appendix*, section 3.3.

The equivalence between probabilities and eigenvalues means that finding the smallest set of eigenvalues that sum to 1−ϵ of the total is equivalent to finding the smallest set of outcomes that accounts for 1−ϵ of the total probability mass of Z. This smallest set of outcomes is sometimes called an ϵ-high-probability set (80). The (1−ϵ)-linear dimension is the size of this high-probability set.

The asymptotic equipartition property (80) guarantees that asymptotically the high-probability set contains $2^{DH(\gamma )}$ outcomes, where $H\left(\gamma \right)=-\sum_{p}\gamma_{p}\log_{2}\gamma_{p}$ is the Shannon entropy of the eigenvalues of each component (normalized to sum to 1). Thus, the linear dimension again grows exponentially with intrinsic dimension (further details in *SI Appendix*, section S3). When tuning along different dimensions has different shapes, the same form holds but with the entropy replaced by the average entropy of the individual factors. Moreover, the linear dimension of data from the multiplicative model grows as the product of linear dimensions of the individual factors. The scaling is asymptotic, but in practice convergence is very rapid, as shown in Fig. 4*D*, *G*, and *J* and *SI Appendix*, Fig. S1.

For a nonasymptotic lower bound on the linear dimension, assume that only two eigenvalues for each factor are nonzero (and thus, the overestimate of the intrinsic dimension of 1 by the linear dimension for each multiplicative factor is as small as possible while still being an overestimate). When normalized to sum to 1, these eigenvalues can be written as {1−γ,γ}, for some γ≤0.5. The eigenvalues of C are again tensor products of {1−γ,γ} taken D times. In descending order of magnitude, there is 1 eigenvalue of magnitude ${\left(1-\gamma \right)}^{D}$, $\left(\begin{array}{c} D\\ 1 \end{array}\right)$ eigenvalues of magnitude ${\left(1-\gamma \right)}^{D-1}\gamma$, and so on, with $\left(\begin{array}{c} D\\ k \end{array}\right)$ eigenvalues of magnitude ${\left(1-\gamma \right)}^{D-k}\gamma^{k}$. Due to the normalization, the sum of the eigenvalues $\sum_{k=0}^{D}\left(\begin{array}{c} D\\ k \end{array}\right){\left(1-\gamma \right)}^{D-k}\gamma^{k}=1$.

These eigenvalues are the outcome probabilities for a binomial random variable X distributed as Bin(D,γ) (i.e., D trials with success probability γ). Thus, $L_{1-\epsilon }$ is the size of the smallest subset of outcomes of a binomial random variable that account for (1−ϵ) of the probability. For ϵ≤0.5, standard lower bounds on sums of binomial coefficients yield

[12] $$\begin{array}{c} L_{1-\epsilon }\geq \frac{1}{\sqrt{8\rho (1-\rho )}}2^{(H_{b}\left(\rho \right)-\frac{\log_{2}\left(D\right)}{2D})D}, \end{array}$$

where $H_{b}\left(\rho \right)=-\rho \log_{2}\rho -\left(1-\rho \right)\log_{2}\left(1-\rho \right)$-pagination

is the binary entropy function and ρ is lower bounded by γ−(1+ln(2))/D (see *SI Appendix*, section 3.3 for full argument). Thus, except when D is small enough that $H_{b}\left(\rho \right)<\frac{\log_{2}\left(D\right)}{2D}$ the lower bound grows exponentially with D [with the exponent asymptotically approaching $H_{b}\left(\gamma \right)$].

In Fig. 4*B*–*J*, we numerically verify the arguments in this section for three examples of multiplicative tuning curves. Fig. 4*B*–*D* shows tuning curves that are products of sigmoidal factors, with sigmoids having a range of slopes and centers. Fig. 4*E*–*G* shows tuning curves that are products of Gaussian factors with different widths and centers. And Fig. 4*H*–*J* shows tuning curves that are products of mixtures of sigmoidal and Gaussian factors. In *SI Appendix*, Fig. S1, we also show results from a model-agnostic setting where eigenvalues for the factors are directly generated using symmetric Dirichlet distributions with different concentration parameters. In all cases, eigenvalues are the tensor product of the eigenvalues of the component matrices (*Center* column), and linear dimension grows exponentially with intrinsic dimension (*Right* column).

To summarize, for multiplicative tuning the linear dimension grows exponentially with intrinsic dimension, with a scaling constant that approaches the average entropy of the eigenvalue distribution for a single factor.

## Discussion

The relationship between intrinsic and linear dimension provides insight into fundamental features of neural information encoding (such as generalizability and the progress of learning) as well as constraints on statistical tools that can be used to analyze data (1, 9, 29). It is widely appreciated that the point clouds and manifolds that emerge from neural population data are often nonlinear (9, 15, 18, 81), and previous work has in particular identified the sparsity of neural population responses as an important factor in this nonlinearity (4, 10, 17, 29). The present study shows that the nonlinearity is likely to be exceedingly high—for a number of common population codes, linear dimension grows at least exponentially with the intrinsic dimension of data. This exponential growth holds even if representations are not sparse; thus, even quite distributed population codes can have extremely high linear dimension. Consequently, dimensionality reduction methods that fit a linear subspace to data, such as PCA and Factor Analysis, will dramatically overestimate the true dimension of data drawn from these population codes.

The analytical results here show exponential or faster growth of linear dimension with intrinsic dimension for both translation-symmetric and multiplicative population codes. These results likely apply more generally to populations of neurons with sparse or localized firing fields on some low-dimensional manifold, even if these firing fields take different shapes across neurons (*SI Appendix*, Fig. S6), as well as to tuning curves that can be approximated by a product of lower-dimensional factors. A particularly important case is populations where tuning curves are approximately translation-symmetric, but there also exist special locations with higher densities of tuning curves or where neurons are more sharply tuned. Examples of this form of approximate translation symmetry include orientation tuning in V1, where cardinal directions are encoded with higher density (82), and hippocampal place cells, which cluster around reward and landmark locations (83). In *SI Appendix*, Fig. S7 we show that adding neurons concentrated at a set of special locations to an otherwise homogeneous population serves to either preserve linear dimension (if the added neurons have the same tuning width as the homogeneous population) or increase linear dimension (if the added neurons have sharper tuning). Thus, the linear dimension of the homogeneous case is a lower bound for the inhomogeneous case.

High linear dimension reflects the structure of the underlying manifolds or point clouds and does not reflect a lack of data or the presence of noise. The results apply in the limit of large amounts of data and number of sampled neurons and in the absence of noise. For finite data, the observed linear dimension may be limited by the number of recorded neurons and the complexity of the task or experimental setting (54) but grow as more neurons and task variable values are measured (*SI Appendix*, Fig. S5). In the presence of noise, the observed linear dimension will be even higher than the noise-free calculations, and thus, the lower bounds will still hold. Finally, while we choose 90 to 95% variance explained as our criterion to define linear dimension for the figures, no results depend on this particular threshold, and exponential growth of linear dimension with intrinsic dimension is required to capture any nonvanishing fraction of variance.

Previous theoretical work measuring linear dimension has focused on the participation ratio (14, 54, 84–86). If $\left\{\lambda_{1},\cdots ,\lambda_{N}\right\}$ are the eigenvalues of the data covariance matrix, then the participation ratio (PR) is ${\left(\sum_{n}\lambda_{n}\right)}^{2}/\sum_{n}\lambda_{n}^{2}$. Thus, if the eigenvalue mass is concentrated on a few large eigenvalues the PR is low. Notably, Recanatesi et al. show that for 2-dimensional Gaussian tuning, the PR increases inversely proportional to tuning curve width, much as we find (29). We instead define linear dimension as the number of eigenvalues required to account for a certain fraction (i.e., 1−ϵ) of total variance, as this is more easily interpretable in terms of data variance, and closely matches what is done in practice when using methods such as PCA (1, 9, 30, 81). Depending on the particular eigenvalue distribution, the PR typically corresponds to the number of eigenvalues required to explain about 80 to 95% of the variance and is thus well-correlated with our definition of linear dimension (54). Moreover, Wigderson and Wigderson (69) derive an uncertainty principle for localization as measured by the PR. In the context of our results, for translation-symmetric tuning, this principle implies that the PR of the eigenvalue distribution must grow as the number of neurons divided by the spread of the covariance profile (similar to the growth of linear dimension with sparsity), where spread of the covariance profile is also measured using PR. Consequently, for translation-symmetric tuning our results will extend to linear dimension as measured by the PR. Similarly, for multiplicative tuning, the PR of a product of factors is equal to the product of the PRs, thus again yielding exponential scaling. More generally, the PR has a number of nice mathematical properties and for these reasons was suggested as a more theoretically tractable alternative to fraction of variance explained when measuring dimensionality of neural population datasets (54). PR may thus provide a useful way to extend these results to other population codes.

The analyses presented here suggest that neural data from many brain regions should appear high-dimensional when viewed through linear dimensionality reduction methods, where “high-dimensional” is to be interpreted as large when compared to the number of encoded variables but still low-dimensional with respect to the number of neurons in a brain region. In accordance with our observations, recent data from large neural population datasets show high linear dimension (4, 87), and recordings from a number of low-dimensional systems appear distorted and significantly higher-dimensional than they actually are when viewed through linear methods as compared to nonlinear methods (15, 17, 18, 70).

Despite these observations, in many settings linear methods such as PCA have been successful at extracting structure from neural population data. What could explain this good performance?

One possibility is that the observed linear dimension is limited by task structure (54). Gao and Ganguli show that the linear dimension (as measured by PR) of neural data is upper-bounded by a measure of task complexity that is low in common neuroscience tasks. In many cases, their measure of task complexity grows exponentially with the number of task parameters. Thus, one test of the hypothesis that linear dimension is indeed limited by task structure is if observed linear dimension grows very rapidly as more task parameters are added. Another possible test is if linear dimension is higher in the case of naturalistic stimuli or during resting state activity when compared to more controlled task conditions, for which there is some evidence (88).

A second explanation is that the nonlinearity of neural point clouds and manifolds differs substantially across brain regions, reflecting differences in coding strategies, as suggested by recent work (9, 29). Our results most naturally apply to sensory coding, to hippocampal circuits that reflect spatial information, and to sparse combinatorial encoding of information in the cognitive cortex. By contrast, if linear decodability reflects generalizability (6) then brain regions that construct generalizable representations may show comparatively low linear dimension. Or, if a brain region acts to transform an initial condition into a particular dynamical pattern of activity, as suggested for motor cortex (89), then the data will be dominated by the linear dimension of the underlying dynamical system. This dynamical system may occupy a low-dimensional linear subspace because of constraints on learning and connectivity structure or the need for smoothness, controllability, and avoiding chaos (3, 9, 12, 22). As a third possibility, confining neural dynamics to low-dimensional linear subspaces that differ across tasks might enable efficient continual learning without interference (90). Thus, as recently proposed the ratio of linear to intrinsic dimension might be a useful signature of encoding strategies and task demands across brain regions and over the course of learning (9, 29). Characterizing this ratio is increasingly tractable given advances in large-scale recordings and manifold learning algorithms.

Methods that seek the intrinsic dimension (D) of a nonlinear data manifold rather than using the dimension of a linear embedding (L) are an active area of research (15, 17, 29, 32, 55, 81, 86, 87, 91–97). One promising set of approaches draws on powerful embedding theorems that show that D-dimensional manifolds can be generically embedded into space of dimension 2D+1, potentially much lower than the linear dimension (98, 99). In particular, the study of Tajima et al. (100) combines delay embedding with random projections to provide a potentially robust and scalable way of estimating intrinsic dimension in neural population data. A second promising approach uses population dynamics to reconstruct distances between manifold states and has shown success on manifolds derived from translation-symmetric tuning (15, 18). These and other approaches may successfully replace linear methods when dealing with highly nonlinear data.

This study identifies a natural class of low-dimensional nonlinear manifolds that should exist in neural data. These manifolds could be a useful theoretically tractable setting to evaluate methods that estimate intrinsic dimension. For example, dimensionality estimation algorithms could be applied to simulated data generated from a population of neurons with Gaussian tuning to a D-dimensional latent variable, with added noise. The algorithms could then be compared on whether they successfully extract these extremely nonlinear manifolds, how efficiently they do so in terms of computation time and samples, and how robust they are to noise.

Finally, while these results suggest caution when applying PCA and other linear methods, they raise the encouraging possibility that, at least in certain brain regions, low-dimensional population structure may have been missed by linear analyses.

## Materials and Methods

### Figure 2.

For Fig. 2 *C* and *D*, there are N=50 neurons uniformly spaced in [0,1] and $N_{\alpha }=10^{4}$ values of the latent variable are drawn from a uniform distribution in [0,1], so that A is a $50\times 10^{4}$ matrix. All neurons have Gaussian tuning curves with a fixed width σ and periodic boundary conditions. In Fig. 2*C*, circles are eigenvalues of the covariance matrix from simulations and lines are theoretical predictions from *SI Appendix*, Eq. **22**. In Fig. 2*D*, circles are $L_{0.95}-1$ calculated from simulations, and the line is theoretical prediction in *SI Appendix*, Eq. **24**. Note that to be conservative with regard to finite size effects, for all numerical simulations we subtract one when plotting the computed linear dimension—this, for example, ensures that if 9 principal components account for 94.9% of the variance then the plotted linear dimension is 9 rather than 10; it also guarantees that lower bounds apply to both non-mean-subtracted and mean-subtracted data.

### Figure 3.

Circles in Fig. 3*C*, *E*, and *F* are calculated from simulations with data generated from D dimensional Gaussian tuning curves with periodic boundary conditions along each dimension with the following parameters: D=2, 10 neurons per dimension (100 total); D=3, 10 neurons per dimension (1,000 total); D=4, 8 neurons per dimension (4,096 total). Simulations used $10^{4}$ uniformly distributed values of the latent variable. In Fig. 3*C*, σ=0.15. Thin lines are eigenvalues from theoretical prediction Eq. **8**. Thick lines are smooth interpolations from Eq. **9**. In Fig. 3 *E* and *F*, diamonds are linear dimensions found numerically from tensor product of 1D eigenvalues (used to speed up computation as D gets larger). Dashed lines are semianalytic approximation found from the chi-distribution. Solid lines are lower bounds from the median of a chi distribution and apply to any $L_{1-\epsilon }$ provided ϵ<0.5 and D>1.

### Figure 4.

For Fig. 4*B*–*D*, neurons along each dimension have sigmoidal response functions $f\left(x,\mu \right)=1/\left(1+e^{-s(x-\mu )}\right)$. For simulations, there are 8 neurons along each dimension with uniformly spaced μ in [0,1]. Slopes s are uniformly spaced in the range [−5,5]. For Fig. 4*E*–*G*, neurons along each dimension have Gaussian tuning curves with varying widths, starting with the minimum value at the center of the range [0,1] and increasing toward the ends. There are 8 neurons along each dimension. The minimum width is 0.05 and increases in steps of 0.05 to the maximum of 0.2. In Fig. 4 *C* and *F*, circles are eigenvalues of covariance matrices calculated from D dimensional data and lines are tensor products of eigenvalues of the 1D covariance matrix, shown in the *Inset*. In Fig. 4 *D* and *G*, circles are $L_{0.9}$ calculated from simulations and lines are asymptotic lower bounds $2^{D(H-0.05)}$ where H is the entropy of the normalized 1D eigenvalues shown in the *Inset* of Fig. 4 *C* and *F*. Note that asymptotically $2^{D(H-\delta )}$ is a lower bound for any δ, so the choice of 0.05 is for convenience but also shows that exponential scaling applies for small D. Fig. 4*H*–*J* show a model that has equal numbers of sigmoidal and Gaussian dimensions (i.e., a hybrid of the models shown in Fig. 4 *B* and *E*).

## Supplementary Material

Appendix 01 (PDF)

## Data Availability

Simulation code is available at https://github.com/chaudhurilab/manifold-lin-dim (101).

## Footnotes

^*^$i=\sqrt{-1}$ throughout this study and is not used as a matrix index.

^†^Note that this problem is closely related to finding a 95% (or any other percent) CI for a D-dimensional Gaussian. While an interval of width 2σ contains 95% of the probability mass in 1D, in higher dimensions an interval of any fixed width contains a shrinking fraction of the total probability mass. Thus the interval must grow with D.
