# Supplementary material for: Common population codes produce extremely nonlinear neural manifolds
Source: Proc Natl Acad Sci U S A. 2023 Sep 21;120(39):e2305853120. doi: 10.1073/pnas.2305853120 (PMC10523500; doi:10.1073/pnas.2305853120)
Supplement: Supplementary file 1 — Appendix 01 (PDF) [file pnas.2305853120.sapp.pdf]

Supplementary Information

Contents

S1 Linear dimension of mean subtracted data is lower bounded by the linear dimension of non-mean-subtracted data minus 1 **1**

S2 Translation-symmetric tuning curves **2**
- S2.1 The covariance matrix of translation-symmetric data is translation-symmetric 2
- S2.2 Eigenvalues of translation-symmetric covariance matrices 4
- S2.3 Gaussian tuning curves show a Gaussian covariance profile 5
- S2.4 Variance explained and linear dimension for one-dimensional Gaussian tuning curves 6
- S2.5 Variance explained and exponential scaling of linear dimension for multi-dimensional Gaussians 7
- S2.6 Supra-exponential growth of linear dimension for localized tuning curves from uncertainty principles 9

S3 Multiplicative tuning curves **9**
- S3.1 Notational preliminaries 10
- S3.2 Covariance matrix and eigenvalues of covariance matrix for multiplicative tuning curves 10
- S3.3 The linear dimension of multiplicative models grows exponentially with intrinsic dimension 12
  - S3.3.1 Factors have same functional form 12
  - S3.3.2 Factors have different functional forms 12
  - S3.3.3 Finite size results and convergence to asymptotic scaling 13

S4 Further numerical experiments **15**

S1 Linear dimension of mean subtracted data is lower bounded by the linear dimension of non-mean-subtracted data minus 1

We consider a population of N neurons with activities at time t given by $\boldsymbol{y}(t) = [y_1(t), y_2(t), \cdots, y_N(t)]$. The mean of the population activity is $\boldsymbol{\mu} = \mathbb{E}_t[\boldsymbol{y}(t)]$, where the expectation value is taken over time. The non-mean subtracted covariance matrix is given by $C = \mathbb{E}_t\left[\boldsymbol{y}\boldsymbol{y}^T\right]$. And the mean-subtracted covariance matrix is given by $T = \mathbb{E}_t\left[(\boldsymbol{y}-\boldsymbol{\mu})(\boldsymbol{y}-\boldsymbol{\mu})^T\right]$. Note that $C = T + \boldsymbol{\mu}\boldsymbol{\mu}^T$.

The matrices C, T and $\boldsymbol{\mu}\boldsymbol{\mu}^T$ are Hermitian and positive semi-definite. Moreover, $\boldsymbol{\mu}\boldsymbol{\mu}^T$ is rank 1, with 1 non-zero eigenvalue $||\boldsymbol{\mu}||^2$ and remaining eigenvalues 0. Let the eigenvalues of these matrices be denoted as follows

$$
\begin{aligned}
C &: \lambda_1 \geq \lambda_2 \geq \cdots \geq \lambda_N \geq 0 \\
T &: \rho_1 \geq \rho_2 \geq \cdots \geq \rho_N \geq 0 \\
\mu\mu^T &: \nu_1 \geq \nu_2 = \cdots = \nu_N = 0
\end{aligned} \tag{1}
$$

The Weyl inequality for Hermitian matrices yields

$$
\rho_a + \nu_b \leq \lambda_p \leq \rho_r + \nu_s \quad a + b - N \geq p \geq r + s - 1 \tag{2}
$$

With $a = p, b = N, r = p-1, s = 2$ we get

$$\rho_p \le \lambda_p \le \rho_{p-1} \quad \text{for } p \ge 2. \tag{3}$$

And with $a = 1, b = N, r = 1, s = 1$ we get

$$\rho_1 \le \lambda_1 \le \rho_1 + ||\mu||^2 \tag{4}$$

Thus we have the eigenvalue bounds

$$0 \le \rho_N \le \lambda_N \le \rho_{N-1} \le \cdots \le \lambda_2 \le \rho_1 \le \lambda_1 \le \rho_1 + ||\mu||^2 \tag{5}$$

Now assume that the $(1-\epsilon)$ linear dimension of the non-mean-subtracted system is L and that L is at least 2. Thus

$$\frac{\sum_{k=1}^{L-1} \lambda_k}{\sum_{k=1}^{N} \lambda_k} < (1-\epsilon) \tag{6}$$

Define $\Delta = \sum_{k=1}^{L-2}(\lambda_k - \rho_k) + \lambda_{L-1}$. Note that $0 \le \Delta \le \lambda_1$ by the above inequalities.

$$\begin{aligned}(1-\epsilon) >& \frac{\sum_{k=1}^{L-1} \lambda_k}{\sum_{k=1}^{N} \lambda_k} \ge \frac{\sum_{k=1}^{L-1} \lambda_k - \Delta}{\sum_{k=1}^{N} \lambda_k - \Delta} = \frac{\sum_{k=1}^{L-2} \rho_k}{\sum_{k=1}^{L-2} \rho_k + \sum_{k=L}^{N} \lambda_k} \\ \ge& \frac{\sum_{k=1}^{L-2} \rho_k}{\sum_{k=1}^{L-2} \rho_k + \sum_{k=L-1}^{N-1} \rho_k} = \frac{\sum_{k=1}^{L-2} \rho_k}{\sum_{k=1}^{N-1} \rho_k} \ge \frac{\sum_{k=1}^{L-2} \rho_k}{\sum_{k=1}^{N} \rho_k}\end{aligned} \tag{7}$$

Thus $\frac{\sum_{k=1}^{L-2} \rho_k}{\sum_{k=1}^{N} \rho_k} < (1-\epsilon)$.

Consequently, if the $(1-\epsilon)$-linear dimension of the non-mean-subtracted matrix C is L, the $(1-\epsilon)$-linear dimension of the mean-subtracted matrix T is at least $L-1$ (also see numerical confirmation in Fig. S2). In general, depending on $||\mu||^2$ the linear dimension of T can be much higher than that of C. Thus, lower bounds on the linear dimension of the non-mean-subtracted case transfer simply to lower bounds on the linear dimension of the mean-subtracted case.

S2 Translation-symmetric tuning curves

In this section, we derive the form of the covariance matrix for translation-symmetric tuning curves in Section S2.1 and show that the eigenvalues are given by the Fourier transform of the covariance profile in Section S2.2. We calculate the covariance matrix and its eigenvalues explicitly for translation-symmetric Gaussian tuning curves in Sections S2.3, S2.4, S2.5. We show that the linear dimension grows exponentially with D for Gaussian tuning curves in Section S2.5. Finally, we use an uncertainty principle relating functions and their Fourier transforms to show that the linear dimension for truly localized tuning curves grows supra-exponentially in Section S2.6.

S2.1 The covariance matrix of translation-symmetric data is translation-symmetric

We assume that the neural response depends on a D-dimensional latent variable, and thus the tuning centers, $\boldsymbol{\alpha_n}$ and the stimulus variables $\boldsymbol{x}_s = \boldsymbol{x}(t_s)$ are in $\mathbb{R}^D$.

The ns-th element of the data matrix is

$$A_{ns} = f(\boldsymbol{x_s}, \boldsymbol{\alpha_n}). \tag{8}$$

Here f is a translation-symmetric tuning curve so that $f(\boldsymbol{x_s}+\boldsymbol{\delta}, \boldsymbol{\alpha_n}+\boldsymbol{\delta}) = f(\boldsymbol{x_s}, \boldsymbol{\alpha_n}) = g(\boldsymbol{x_s}-\boldsymbol{\alpha_n})$ for some function g. We assume periodic boundary condition along each dimension with period 1 and thus $\boldsymbol{x}, \boldsymbol{\alpha} \in [0,1]^D$. The latent variable is assumed to be uniformly distributed so that values are drawn uniformly from $[0,1]^D$.

The neuron-neuron covariance matrix is given by

$$\begin{aligned} C_{mn} &= \frac{1}{N_x} AA^T \\ &= \frac{1}{N_x} \sum_s f(\boldsymbol{x_s}, \boldsymbol{\alpha_m}) f(\boldsymbol{x_s}, \boldsymbol{\alpha_n}) \end{aligned} \tag{9}$$

where N_x is the number of latent variable values. In the limit of large N_x this sample mean will converge to $\mathbb{E}[f(\boldsymbol{x}, \boldsymbol{\alpha_m}) f(\boldsymbol{x}, \boldsymbol{\alpha_n})]_{\boldsymbol{x}}$, where the expectation is taken over the latent variable distribution. Thus the covariance matrix can also be written as

$$C_{mn} \approx \int_{\mathcal{S}} d\boldsymbol{x}\, p(\boldsymbol{x}) f(\boldsymbol{x}, \boldsymbol{\alpha_m}) f(\boldsymbol{x}, \boldsymbol{\alpha_n}) = \int_{\mathcal{S}} d\boldsymbol{x}\, f(\boldsymbol{x}, \boldsymbol{\alpha_m}) f(\boldsymbol{x}, \boldsymbol{\alpha_n}) \tag{10}$$

where $p(\boldsymbol{x})$ is the distribution over the latent variable, which we assume to be uniform, and the integral $\int_{\mathcal{S}} d\boldsymbol{x} = \int_{\mathcal{S}} dx_1 \cdots dx_D$, is over all points $\boldsymbol{x}$ in the region $\mathcal{S} = [0,1]^D$. Since the tuning curves are translation-symmetric we have

$$\begin{aligned} C_{mn} &= \int_{\mathcal{S}} d\boldsymbol{x}\, f(\boldsymbol{x}, \boldsymbol{\alpha_m}) f(\boldsymbol{x}, \boldsymbol{\alpha_n}) \\ &= \int_{\mathcal{S}} d\boldsymbol{x}\, f(\boldsymbol{x}-\boldsymbol{\alpha_n}, \boldsymbol{\alpha_m}-\boldsymbol{\alpha_n}) f(\boldsymbol{x}-\boldsymbol{\alpha_n}, 0) \\ &= \int_{\mathcal{S}-\boldsymbol{\alpha_n}} d\boldsymbol{x}'\, f(\boldsymbol{x}', \boldsymbol{\alpha_m}-\boldsymbol{\alpha_n}) f(\boldsymbol{x}', 0) \\ &= \int_{\mathcal{S}} d\boldsymbol{x}\, f(\boldsymbol{x}, 0) f(\boldsymbol{x}, \boldsymbol{\delta}) \\ &= c(\boldsymbol{\delta}) = c(\boldsymbol{\alpha_m}-\boldsymbol{\alpha_n}). \end{aligned} \tag{11}$$

In going from the first to the second line, we have used the translation symmetry of the tuning curves $(f(\boldsymbol{x}, \boldsymbol{\alpha}) = f(\boldsymbol{x}-\boldsymbol{\gamma}, \boldsymbol{\alpha}-\boldsymbol{\gamma}))$; in going from the second line to the third line we have made a change of variable, $\boldsymbol{x}' = \boldsymbol{x}-\boldsymbol{\alpha_n}$ and defined $\boldsymbol{\delta} = \boldsymbol{\alpha_m}-\boldsymbol{\alpha_n}$; and in going from the third line to fourth line we have used the periodic boundary conditions to shift the integral over $\mathcal{S}-\boldsymbol{\alpha_m}$ to $\mathcal{S}$. c is a periodic function of the difference in tuning curve centers with period 1 along each dimension. Thus the covariance between two neurons just depends on the difference between their tuning curve centers.

In the one-dimensional case ($D = 1$), if we take the tuning curve centers to be equally spaced lattice points (i.e., $\alpha_n = n/N$ for $n = 0, \cdots N-1$), then the covariance matrix is circulant meaning that each row is a shifted version of the row above.

$$\begin{aligned} C_{m+1,n} &= c(\alpha_{m+1}-\alpha_n) \\ &= c((\alpha_{m+1}-1/N)-(\alpha_n-1/N)) \\ &= c(\alpha_m-\alpha_{n-1}) \\ &= C_{m,n-1} \end{aligned} \tag{12}$$

Note that this holds for $m, n = 1, \cdots N$, since the periodic boundary conditions allow us to define $C_{N+1,n} = C_{1,n}$ and $C_{m,0} = C_{m,N}$.

Finally, note that the results approximately hold if the boundary conditions are hard rather than periodic, provided that the tuning curves are not too wide. In this case, the deviations from perfect translation-invariance caused by the boundary conditions will be mild and restricted to a small subset of neurons (see Section S4).

S2.2 Eigenvalues of translation-symmetric covariance matrices

It is well-known that matrices with translation-symmetric structure have eigenvalues that are given by the Fourier transform of the function that generates the matrix (i.e., c) [1, 2]. We briefly review those results here.

First, consider tuning curve centers that tile the latent space, forming the points of a lattice with N_d tuning curve centers along the d-th dimension. That is, tuning curve centers along the dth dimension are spaced at intervals $1/N_d$, and take values in $\{1/N_d, 2/N_d, \cdots, 1\}$. There are $N = \prod_d N_d$ neurons in total, spanning all possible combinations of one choice of center for each dimension, and thus covering the total space of $[0,1]^D$. The tuning curve parameter vector for the nth neuron is $\boldsymbol{\alpha_n}$, which has dth component $\alpha_n^d \in \{1/N_d, 2/N_d, \cdots, 1\}$, corresponding to the choice of center for that neuron along dimension d.

Recall that the covariance matrix has entries $C_{mn} = c(\boldsymbol{\alpha_m} - \boldsymbol{\alpha}_n)$, where c has period 1 along each dimension. To find the eigenvalues of C (which we will index by p), find consider the set of N vectors $\boldsymbol{k_p}$ given by

$$\boldsymbol{k_p} = 2\pi(\kappa_p^1, \cdots, \kappa_p^D), \tag{13}$$

where each $\kappa_p^d \in \{1, \cdots, N_d\}$ (and as with the tuning curve centers, the $\boldsymbol{k_p}$ vectors range across all possible combinations of components so that p ranges from 1 to $N = \prod_d N_d$). Corresponding to each $\boldsymbol{k_p}$, define the vector $\boldsymbol{v_p} = (e^{i\boldsymbol{k_p}\cdot\boldsymbol{\alpha_1}}, \cdots, e^{i\boldsymbol{k_p}\cdot\boldsymbol{\alpha_N}})$. Multiplying this vector by the matrix C yields

$$\begin{aligned}(C\boldsymbol{v_p})_m &= \sum_{n=1}^{N} c(\boldsymbol{\alpha_m} - \boldsymbol{\alpha_n}) e^{i\boldsymbol{k_p}\cdot\boldsymbol{\alpha_n}} \\ &= \left(\sum_{n=1}^{N} c(\boldsymbol{\alpha_m} - \boldsymbol{\alpha_n}) e^{-i\boldsymbol{k_p}\cdot(\boldsymbol{\alpha_m}-\boldsymbol{\alpha_n})}\right) e^{i\boldsymbol{k_p}\cdot\boldsymbol{\alpha_m}} \\ &= \left(\sum_{n=1}^{N} c(\boldsymbol{\delta_n}) e^{-i\boldsymbol{k_p}\cdot\boldsymbol{\delta_n}}\right) e^{i\boldsymbol{k_p}\cdot\boldsymbol{\alpha_m}}\end{aligned} \tag{14}$$

Here we have defined $\boldsymbol{\delta_n} = \boldsymbol{\alpha_m} - \boldsymbol{\alpha_n}$ (note that $\boldsymbol{\delta_n}$ also depends on m). As a consequence of the uniform spacing of tuning curve centers and periodic boundary conditions, as n changes $\boldsymbol{\delta_n}$ ranges over all possible tuning parameters. Thus, the sum over n in parentheses is independent of both m and n and we can define $\lambda_p = \sum_{n=1}^{N} c(\boldsymbol{\delta_n}) e^{-i\boldsymbol{k_p}\cdot\boldsymbol{\delta_n}}$. Consequently, $\boldsymbol{v_p}$ is an eigenvector of C with eigenvalue λ_p. Note that λ_p is the term with frequency $\boldsymbol{k_p}$ of the discrete Fourier transform of c.

In particular, for the one-dimensional case, the tuning curve parameter for the nth neuron, α_n, is simply a scalar equal to n/N and the frequency parameter k_p is also a scalar equal to $2\pi p$ (where both n and p range from 1 to N). $\boldsymbol{v_p} = (e^{2\pi i p/N}, \cdots, e^{2\pi i p})$ is an eigenvector with eigenvalue $\lambda_p = \sum_{n=1}^{N} c(n/N) e^{-2\pi i p\, n/N}$.

Alternatively, if tuning curve centers are uniformly distributed through the latent space, either evenly spaced or randomly chosen, then in the large N limit we can consider the translation invariant kernel $c(\boldsymbol{\alpha_m} - \boldsymbol{\alpha_n})$, which is a continuous function of $\boldsymbol{\alpha_m}, \boldsymbol{\alpha_n} \in \mathbb{R}^D$. This is the continuous generalisation of the covariance matrix for translation-symmetric tuning curves derived above. The

product of the matrix C with a vector can then be approximated by the convolution of the kernel with a function $f_1(\boldsymbol{\alpha_n})$,

$$f_2(\boldsymbol{\alpha_m}) = \int d\boldsymbol{\alpha_n}\, c(\boldsymbol{\alpha_m} - \boldsymbol{\alpha_n})\, f_1(\boldsymbol{\alpha_n}) \tag{15}$$

where $\int d\boldsymbol{\alpha_n}$ means an integral over all D dimensional vectors $\boldsymbol{\alpha}_n$. $f(\boldsymbol{\alpha}_n)$ is an eigenfunction if

$$\int d\boldsymbol{\alpha_n}\, c(\boldsymbol{\alpha_m} - \boldsymbol{\alpha_n})\, f(\boldsymbol{\alpha_n}) = \lambda f(\boldsymbol{\alpha_m}) \tag{16}$$

Consider $f(\boldsymbol{\alpha}_n) = e^{i\boldsymbol{k}.\boldsymbol{\alpha}_n}$ where $\boldsymbol{k}$ is an arbitrary D dimensional vector.

$$\begin{aligned} &\int d\boldsymbol{\alpha_n}\, c(\boldsymbol{\alpha_m} - \boldsymbol{\alpha_n})\, e^{i\boldsymbol{k}.\boldsymbol{\alpha_n}} \\ =&\int d\boldsymbol{\alpha_n}\, c(\boldsymbol{\alpha_m} - \boldsymbol{\alpha_n})\, e^{-i\boldsymbol{k}.(\boldsymbol{\alpha_m}-\boldsymbol{\alpha_n})}\, e^{i\boldsymbol{k}.\boldsymbol{\alpha_m}} \\ =&\left(\int d\boldsymbol{\delta}\, c(\boldsymbol{\delta})\, e^{-i\boldsymbol{k}.\boldsymbol{\delta}}\right) e^{i\boldsymbol{k}.\boldsymbol{\alpha_m}} \end{aligned} \tag{17}$$

As before in going from line 2 to line 3, we have defined a new variable, $\boldsymbol{\alpha_m} - \boldsymbol{\alpha_n} = \boldsymbol{\delta}$ and used the periodic boundary conditions to replace the integral over $\boldsymbol{\alpha_n}$ with an integral over $\boldsymbol{\delta}$. The expression in parenthesis does not depend on $\boldsymbol{\alpha_m}$ or $\boldsymbol{\alpha_n}$ and is the Fourier transform of $c(\boldsymbol{\delta})$. So $e^{i\boldsymbol{k}.\boldsymbol{\alpha_n}}$ is an eigenfunction of $c(\boldsymbol{\alpha_m} - \boldsymbol{\alpha_n})$ with eigenvalue $\int d\boldsymbol{\delta}\, c(\boldsymbol{\delta})\, e^{-i\boldsymbol{k}.\boldsymbol{\delta}}$.

Finally, while these results are for uniformly distributed data with periodic boundary conditions, note that covariance matrices are symmetric and thus normal. Consequently, the effect of perturbations on the eigenvalue spectrum is as mild as possible, and the results should hold approximately for matrices that only approximately satisfy these conditions (see Section S4).

S2.3 Gaussian tuning curves show a Gaussian covariance profile

Consider the case of Gaussian tuning, where the tuning curve of the n-th neuron is given by

$$y_n(t) = f(\boldsymbol{x(t)}, \boldsymbol{\alpha_n}) = K_1 \exp\left(-\frac{1}{2}(\boldsymbol{x} - \boldsymbol{\alpha_n})^T \Sigma^{-1} (\boldsymbol{x} - \boldsymbol{\alpha_n})\right). \tag{18}$$

Here Σ is the covariance matrix of the tuning curve.

Note that Gaussians are defined on an infinite rather than circular interval. Thus, assuming that tuning curves are well-described by Gaussians requires that they are not too wide, so that the firing rate of a neuron centered at the middle of the interval decays to 0 by the ends of the interval. This is a mild requirement, equivalent to requiring that no neuron responds significantly to the entire range of latent variable values. However, the results presented in the previous section relating covariance profile to eigenvalue spectrum are general, as are the corresponding uncertainty principle-derived relationships between covariance profile and linear dimension, and do not require any particular form of the tuning curves. Thus the theory can be extended to wider tuning curves using a different functional form (e.g., von Mises functions) at the expense of simple closed form expressions for linear dimension (note that separately, in Section S2.5, we show that for Gaussian tuning curves exponential growth of linear dimension does not apply if tuning curves are above a certain threshold width).

To generate the neuron-neuron covariance matrix, we wish to calculate the covariance profile $c(\boldsymbol{\delta}) = \int_{\mathcal{S}} d\boldsymbol{x}\, f(\boldsymbol{x}, 0) f(\boldsymbol{x}, \boldsymbol{\delta})$, which is the covariance between pairs of neurons whose tuning curve centers are separated by $\boldsymbol{\delta}$. Note that c is periodic, with period 1 in each direction. For notational

convenience we shift the range of $\boldsymbol{\delta}$ so that each component lies within $[-1/2, 1/2]$ rather than $[0, 1]$. Thus,

$$
\begin{aligned}
c(\boldsymbol{\delta}) &= \int_{\mathcal{S}} d\boldsymbol{x}\, f(\boldsymbol{x}, 0) f(\boldsymbol{x}, \boldsymbol{\delta}) \\
&= K_1^2 \int_{\mathcal{S}} \boldsymbol{dx} \exp\left(-\frac{1}{2}\boldsymbol{x}^T \Sigma^{-1} \boldsymbol{x}\right) \exp\left(-\frac{1}{2}(\boldsymbol{x}-\boldsymbol{\delta})^T \Sigma^{-1} (\boldsymbol{x}-\boldsymbol{\delta})\right) \\
&= K_1^2 \int_{\mathcal{S}} \boldsymbol{dx} \exp\left[-\left(\boldsymbol{x}^T \Sigma^{-1} \boldsymbol{x} - \frac{\boldsymbol{\delta}^T \Sigma^{-1} \boldsymbol{x}}{2} - \frac{\boldsymbol{x}^T \Sigma^{-1} \boldsymbol{\delta}}{2} + \frac{\boldsymbol{\delta}^T \Sigma^{-1} \boldsymbol{\delta}}{2}\right)\right] \\
&= K_1^2 \int_{\mathcal{S}} \boldsymbol{dx} \exp\left[-\left(\boldsymbol{x} - \frac{\boldsymbol{\delta}}{2}\right)^T \Sigma^{-1} \left(\boldsymbol{x} - \frac{\boldsymbol{\delta}}{2}\right)\right] \exp\left(-\frac{\boldsymbol{\delta}^{\boldsymbol{T}} \Sigma^{-1} \boldsymbol{\delta}}{4}\right) \\
&= K_1^2 \exp\left(-\frac{\boldsymbol{\delta}^{\boldsymbol{T}} \Sigma^{-1} \boldsymbol{\delta}}{4}\right) \int_{\mathcal{S}} \boldsymbol{dx} \exp\left[-\left(\boldsymbol{x} - \frac{\boldsymbol{\delta}}{2}\right)^T \Sigma^{-1} \left(\boldsymbol{x} - \frac{\boldsymbol{\delta}}{2}\right)\right] \\
&= K_2 \exp\left(-\frac{\boldsymbol{\delta}^{\boldsymbol{T}} \Sigma^{-1} \boldsymbol{\delta}}{4}\right).
\end{aligned}
\tag{19}
$$

Here the constant $K_2 = K_1^2 \sqrt{\det(\pi\Sigma)}$. Thus, neurons with Gaussian tuning curves have a Gaussian covariance profile with width twice that of the tuning curve.

S2.4 Variance explained and linear dimension for one-dimensional Gaussian tuning curves

In the one-dimensional case, the covariance profile is

$$
\begin{aligned}
c(\delta) =& K_2 \exp\left(-\frac{\delta^2}{4\sigma^2}\right), \quad \delta \leq \frac{1}{2} \\
=& K_2 \exp\left(-\frac{(1-\delta)^2}{4\sigma^2}\right), \quad \delta > \frac{1}{2}
\end{aligned}
\tag{20}
$$

If tuning curve centers are evenly spaced, this covariance profile is sampled at $\delta_n = n/N$, where n takes values in $[0, \dots, N-1]$.

As described in Section S2.2, the eigenvalues of the covariance matrix are given by the Fourier transform of this profile. For convenience we index the eigenvalues by p ranging from $\lfloor(-N+1)/2\rfloor$ to $\lfloor N/2\rfloor$. Ignoring the overall normalization constant K_2, for each such p we have an eigenvalue

$$
\begin{aligned}
\lambda_p &= \sum_{n=0}^{N-1} c(n/N) e^{-2\pi i p\, n/N} = \sum_{n=0}^{\lfloor N/2\rfloor} c(n/N) e^{-2\pi i p\, n/N} + \sum_{l=1}^{\lfloor (N+1)/2\rfloor} c(1-l/N) e^{2\pi i p\, l/N} \\
&= \sum_{n=0}^{\lfloor N/2\rfloor} \exp\left(\frac{-n^2}{4\sigma^2 N^2}\right) \exp\left(\frac{-2\pi i n p}{N}\right) + \sum_{l=\lfloor(-N+1)/2}^{-1} \exp\left(\frac{-l^2}{4\sigma^2 N^2}\right) \exp\left(\frac{2\pi i l p}{N}\right) \\
&= \sum_{n=\lfloor(-N+1)/2\rfloor}^{\lfloor N/2\rfloor} \exp\left(\frac{-n^2}{4\sigma^2 N^2}\right) \exp\left(\frac{-2\pi i n p}{N}\right) \\
&= K_3 \exp\left(-4\pi^2\sigma^2 p^2\right)
\end{aligned}
\tag{21}
$$

where K_3 is a constant, and in the last line we have completed the square and noted that the sum over n is a constant. Thus the eigenvalues have a Gaussian profile with width $\frac{2\sqrt{2}\pi}{\sigma}$. Also note

that the eigenvalues decrease monotonically with the magnitude of p and that, except for λ_0, they occur in pairs with $\lambda_p = \lambda_{-p}$.

Since the eigenvalues occur in pairs, the $(1-\epsilon)$-linear dimension is the smallest $L_{1-\epsilon}$ such that

$$\sum_{p=0}^{\frac{L_{1-\epsilon}}{2}} \lambda_p \geq (1-\epsilon) \sum_{p=0}^{N/2} \lambda_p. \tag{22}$$

For large N, approximating both sides of this equation as an integral and canceling the common prefactor K_3 yields

$$\begin{aligned}
\int_0^{\frac{L_{1-\epsilon}}{2}} e^{-(2\pi\sigma p)^2} dp \geq\ & (1-\epsilon) \int_0^{\infty} e^{-(2\pi\sigma p)^2} dp = \frac{(1-\epsilon)}{4\sqrt{\pi}\sigma} \\
4\sqrt{\pi}\sigma \int_0^{\frac{L_{1-\epsilon}}{2}} e^{-(2\pi\sigma p)^2} dp \geq\ & (1-\epsilon) \\
\frac{2}{\sqrt{\pi}} \int_0^{\pi\sigma L_{1-\epsilon}} e^{-z^2} dz \geq\ & (1-\epsilon) \\
\operatorname{erf}(\pi\sigma L_{1-\epsilon}) \geq\ & (1-\epsilon) \\
L_{1-\epsilon} =\ & \frac{1}{\sigma} \frac{\operatorname{erf}^{-1}(1-\epsilon)}{\pi}
\end{aligned} \tag{23}$$

Thus, $L_{1-\epsilon}$ grows as $\frac{1}{\sigma}$, with a proportionality constant that depends on the fraction of variance explained. In particular, for 95% variance explained, we have $L_{0.95} = \frac{1.96}{\sqrt{2}\,\pi\sigma}$.

S2.5 Variance explained and exponential scaling of linear dimension for multi-dimensional Gaussians

We next consider a population neurons with Gaussian tuning to an underlying D-dimensional latent variable. We assume that the tuning curve centers lie on a D dimensional grid with N_D equally spaced centers along each dimension. Therefore there are $N = N_D^D$ neurons.

For simplicity we also assume that the width of the tuning is the same along each direction so that the covariance matrix of the tuning curve Σ is diagonal with elements σ^2 along the diagonal. Thus, as shown in Sec S2.2, the covariance between two neurons whose tuning curve centers are separated by $\boldsymbol{\delta}$ is $c(\boldsymbol{\delta}) = K_2 \exp\left(-\frac{||\boldsymbol{\delta}||^2}{4\sigma^2}\right)$, for $\boldsymbol{\delta} \in [-\frac{1}{2}, \frac{1}{2}]^D$, where K_2 is an overall scaling constant. $c(\boldsymbol{\delta})$ is extended periodically outside this range.

As for the one-dimensional case and as discussed in Sec. S2.2, the eigenvalues of the neuron-neuron covariance matrix are given by the Fourier transform of $c(\boldsymbol{\delta})$. We can index the eigenvalues by a D-dimensional vector $\boldsymbol{p}$ with dth entry $p_d \in [-N_d/2, \cdots, N_d/2]$ yielding

$$\lambda_{\boldsymbol{p}} = K_3 \exp\left(-4\pi^2 \sum_{d=1}^{D} p_d^2 \sigma^2\right) = K_3 \exp(-4\pi^2\sigma^2 ||\boldsymbol{p}||^2). \tag{24}$$

The magnitude of an eigenvalue is thus a function only of $||\boldsymbol{p}||$, the Euclidean distance from the origin in $\boldsymbol{p}$-space. As there are multiple lattice points with the same distance from the origin, there will be multiple eigenvalues with the same magnitude. Moreover, the number of eigenvalues at a given distance from the origin increases as the distance increases. Consequently, as the distance from the origin increases, there will be more eigenvalues but with smaller magnitude.

Overloading notation to define the D-dimensional continuous Gaussian function $\lambda : \mathbb{R}^D \to \mathbb{R}$ as $\lambda(||\boldsymbol{p}||) = K_3 \exp(-4\pi^2\sigma^2||\boldsymbol{p}||^2)$, observe that the eigenvalues are given by $\lambda(||\boldsymbol{p}||)$ sampled at the integer lattice points of a D-dimensional cube with side length N_d.

To estimate the $(1-\epsilon)$ linear dimension, note that summing up the $L_{1-\epsilon}$ largest eigenvalues is equivalent to summing up all eigenvalues for which $||\boldsymbol{p}|| \le R$ (i.e., eigenvalues corresponding to all lattice points with a ball of radius R), up to some edge effects resulting from the discreteness of the lattice points that will vanish when we take the continuous limit below. Thus we first compute the smallest radius R such that

$$\sum_{||\boldsymbol{p}|| \le R} \lambda_{\boldsymbol{p}} \ge (1-\epsilon) \sum_{p} \lambda_{\boldsymbol{p}}. \tag{25}$$

Approximating the sum by the integral of $\lambda(\boldsymbol{p})$ and setting $K_5 = \int_{||\boldsymbol{p}||} d\boldsymbol{p}\lambda(\boldsymbol{p})$, we seek R such that

$$\frac{1}{K_5} \int_{||\boldsymbol{p}|| \le R} dp_1 dp_2 \cdots dp_D \exp\left(-(2\pi\sigma)^2 \sum_{d=1}^{D} p_d^2\right) \ge (1-\epsilon). \tag{26}$$

Converting to radial coordinates with $r^2 = \sum_{d=1}^{D} p_d^2$ and then making a second change of coordinates $y = \sqrt{8}\pi\sigma r$ yields

$$\begin{aligned} \frac{1}{K_5} \int_{||\boldsymbol{p}|| \le R} dp_1 \ldots dp_D \exp\left(-(2\pi\sigma)^2 \sum_{d=1}^{D} p_d^2\right) &= \frac{1}{K_6} \int_0^R dr\, r^{D-1} \exp\left(-(2\pi\sigma)^2 r^2\right) \\ &= \frac{1}{K_7} \int_0^{\sqrt{8}\pi\sigma R} dy\, y^{D-1} \exp\left(-y^2/2\right), \end{aligned} \tag{27}$$

where $K_5 = \left(\frac{\pi}{(2\pi\sigma)^2}\right)^{D/2}$, $K_6 = \frac{\Gamma(D/2)}{2(4\pi^2\sigma^2)^{D/2}}$ and $K_7 = 2^{(D/2)-1}\Gamma(D/2)$.

Note that λ is the (unnormalized) probability density function of a multivariate Gaussian distribution and the argument of the integral above is the density function of a chi distribution with D degrees of freedom (appropriately normalized as a result of dividing by the integral across the entire range). Thus, defining the random variable Z distributed according to a chi distribution with D degrees of freedom, note that the integral itself corresponds to the CDF of Z and we seek the smallest R such that $P(Z \le \sqrt{8}\pi\sigma R) \ge 1-\epsilon$.

If $\epsilon < 1/2$ (i.e., we are capturing at least 50% of the variance in the data), then by definition R must be at least the median value of Z. Standard results on chi distributions then show that $\sqrt{8}\pi\sigma R \ge \sqrt{D}\left(1 - \frac{2}{9D}\right)^{3/2} > 0.8\sqrt{D}$, where the final inequality holds for $D \ge 2$. Consequently, $R > \frac{0.8}{\sqrt{8}\pi}\frac{\sqrt{D}}{\sigma}$. Note that as D gets larger the factor of 0.8 in the lower bound can be strengthened to a factor of 1.

Finally, to estimate the linear dimension itself we need to count the number of eigenvalues that lie within a sphere of radius R. Since the eigenvalues correspond to lattice points, the number of eigenvalues is approximately the volume of this D-dimensional sphere yielding

$$L_{1-\epsilon} \approx V_D = \frac{\pi^{D/2}}{\Gamma\left(\frac{D}{2}+1\right)} R^D > \frac{1}{\sqrt{D\pi}} \left(\frac{0.4\sqrt{e}}{\sigma\sqrt{\pi}}\right)^D = \frac{1}{\sqrt{D\pi}} \left(\frac{K_4}{\sigma}\right)^D \tag{28}$$

where K_4 is a constant, and we have used Stirling's approximation for the gamma function. If $\sigma < K_4$ then the quantity in parentheses is > 1 and thus this lower bound increases exponentially. Consequently, the linear dimension grows exponentially with D as long as σ is not too large (note that the range along each dimension is normalized to $[0, 1]$, and the threshold on σ effectively means that single tuning curves do not span the entire range of stimulus values). We show results for wide tuning curves and the onset of exponential scaling in Fig. S4.

S2.6 Supra-exponential growth of linear dimension for localized tuning curves from uncertainty principles

In this section we consider genuinely localized tuning curves, by which we mean tuning curves that are contained within some finite support rather than having small but infinite tails (i.e., unlike the Gaussian tuning curves in the previous section). We then use the uncertainty principles of Wigderson & Wigderson [3] to show that in this case linear dimension grows supra-exponentially with D.

We assume that the neurons have N_D equally spaced tuning curve centers along each dimension in the hypercube $[-1/2, 1/2]^D$. Thus, there are a total of N_D^D neurons in a volume of $\mathbf{1}^D$. If tuning curves have finite support, then the tuning curve of a neuron is contained within some fixed radius $R/2$. We assume that an individual tuning curve does not cover more than half the space, so that $R/2 < 1/4$. Consequently, the covariance between two neurons is 0 unless the centers of their firing fields lie within a distance $R < 1/2$. Thus,

$$\begin{aligned} c(\boldsymbol{\alpha_m} - \boldsymbol{\alpha_n}) = c(\boldsymbol{\delta}) &\geq 0, \ \ |\boldsymbol{\delta}| \leq R \\ &= 0, \ \ |\boldsymbol{\delta}| > R \end{aligned} \tag{29}$$

The support of the covariance profile c is thus upper-bounded by the number of points inside a sphere of radius R. Since there are N_D^D points in a volume of 1^D, the support of the covariance profile c is,

$$\begin{aligned} |supp(c)| \leq & Vol_D(R) \times N_D^D \\ = & \frac{\pi^{D/2}}{\Gamma(D/2+1)} R^D N_D^D \sim \frac{1}{\sqrt{D\pi}} \left(\frac{2\pi e}{D}\right)^{D/2} (RN_D)^D \end{aligned} \tag{30}$$

As before, the eigenvalues of the covariance profile are given by the Fourier transform of the covariance profile c. Let the eigenvalue profile be λ, with ϵ-support $supp^\epsilon(\lambda)$. From the uncertainty principle for ϵ-support [3], we have

$$\begin{aligned} |supp(c)||supp^\epsilon(\lambda)| \geq & N_D^D(1-\epsilon) \\ |supp^\epsilon(\lambda)| \geq & \frac{N_D^D(1-\epsilon)}{|supp(c)|} \\ |supp^\epsilon(\lambda)| \geq & (1-\epsilon)\sqrt{D\pi} \left(\frac{D}{2\pi e}\right)^{D/2} \left(\frac{1}{R}\right)^D \end{aligned} \tag{31}$$

As before, the size of the ϵ-support of λ is the $1-\epsilon$ linear dimension. Thus, for translation-symmetric tuning curves which are localized within a fixed radius, the linear dimension grows faster than exponentially as $\left(\sqrt{D}\right)^D$.

S3 Multiplicative tuning curves

In this section we show that for multiplicative tuning curves the D dimensional covariance matrix and its eigenvalues can be expressed as a tensor product of 1D covariance matrices and product of 1D eigenvalues respectively. We then find a lower bound on the linear dimension of data from such tuning curves using arguments from probability and information theory.

S3.1 Notational preliminaries

It will be convenient to define some notation to help streamlines indices.

Given a number N, let $[N] = \{1, \ldots, N\}$ be the set of numbers between 1 and N.

Given a set of numbers $\{N_1, \ldots, N_D\}$, let $\Omega = [N_1] \times \ldots \times [N_D]$ be the Cartesian product of the sets $[N_d]$. Thus Ω consists of all D-tuples with the dth element of each tuple chosen from $[N_d]$.

Define $\Phi : \Omega \to [\prod_{d=1}^{D} N_d]$ to be the index of each element in Ω when they are ordered lexicographically. That is, $\Phi((1, \ldots, 1)) = 1$, $\Phi((1, \ldots, 2)) = 2$, and so on up to $\Phi((N_1, \ldots, N_D)) = \prod_{d=1}^{D} N_d$.

Let Ψ be the inverse mapping of Φ. That is, $\Psi(1) = (1, \ldots, 1)$ and so on up to $\Psi(\prod_d N_d) = (N_1, \ldots, N_D)$. And let Ψ^d be the dth component of Ψ.

Consider a set of matrices $\{A^1, \ldots, A^D\}$, with the dimension of the dth matrix being $N_d \times N_d$ (note that the superscripts index the matrices and are not powers). Let $A = A^1 \otimes \ldots \otimes A^D$ be their tensor product as represented in matrix form by the Kronecker product. By the definition of the Kronecker product we have

$$A_{mn} = A^1_{\Psi^1(m)\Psi^1(n)} \cdots A^D_{\Psi^D(m)\Psi^D(n)} = \prod_{d=1}^{D} A^d_{\Psi^d(m)\Psi^d(n)} \tag{32}$$

S3.2 Covariance matrix and eigenvalues of covariance matrix for multiplicative tuning curves

We consider neural tuning curves that are a product of lower-dimensional factors. If $\boldsymbol{x}$ is the latent variable and $\boldsymbol{\alpha}_n$ is the vector of parameters for the nth neuron, we can write the tuning curve of the n-th neuron as

$$y_n(t) = f(\boldsymbol{x}(t), \boldsymbol{\alpha}_n) = \prod_{d=1}^{D} f_d(x^d(t), \alpha_n^d). \tag{33}$$

Here the superscript denotes the d-th component of a vector, and the f_d's are scalar functions. For simplicity, we assume that each multiplicative factor f_d is a function of a one-dimensional variable x^d (i.e., the dth component of $\boldsymbol{x}$). However in general the f_d's could be functions of disjoint sets of multiple variables and similar results hold.

Assuming that the distribution of the latent variable along each dimension is independent (i.e., $p(\boldsymbol{x}) = \prod_d p(x^d)$) and rectangular boundary conditions, the covariance profile between neurons with tuning parameters $\boldsymbol{\alpha_m}$ and $\boldsymbol{\alpha_n}$ is

$$\begin{aligned} c(\boldsymbol{\alpha_m}, \boldsymbol{\alpha_n}) &= \int d\boldsymbol{x}\, p(\boldsymbol{x}) f(\boldsymbol{x}, \boldsymbol{\alpha_m}) f(\boldsymbol{x}, \boldsymbol{\alpha_n}) \\ &= \prod_{d=1}^{D} \int dx^d\, p(x^d) f_d(x^d, \alpha_m^d) f_d(x^d, \alpha_n^d) \\ &\equiv \prod_{d=1}^{D} c^d(\alpha_m^d, \alpha_n^d), \end{aligned} \tag{34}$$

where the last line serves to define the individual covariance factors c^d.

The function c provides the covariance between any two neurons, while each function c^d provides the portion of the covariance that comes from the similarity of responses along the dth dimension.

We next choose the locations of the tuning curve parameters to tile the space, forming the points of a lattice with N_d tuning curve parameters along the dth dimension. Let the parameters

along the d-th dimension be $\left\{\beta_1^d, \ldots, \beta_{N_d}^d\right\}$. Note that these parameters do not need to be equally spaced.

Since the neuron parameters tile the space, there are totally $N = \prod_d N_d$ neurons in the population and the D-dimensional parameter vectors for the neurons in the population consist of all possible combinations of one entry from each of these parameter sets. Thus, each $\alpha_m^d \in \left\{\beta_1^d, \ldots, \beta_{N_d}^d\right\}$. For example, for the first neuron we have $\boldsymbol{\alpha_1} = (\beta_1^1, \ldots, \beta_1^D)$ and so on. Adopting the notation from Section S3.1, the corresponding parameter indices for the mth neuron are given by $\Psi(m)$. That is $\alpha_m^d = \beta_{\Psi^d(m)}^d$.

The resulting covariance matrix C has dimension $N \times N$, where $N = \prod_d N_d$. Note that while the function c encodes the covariance between any two neurons, the matrix C contains the covariances for a particular set of neurons sampled from the population (i.e., the function c evaluated for these particular neurons).

We next define a set of smaller matrices C^d of dimension. Let the (r, s) entry of C^d be $C^d(r, s) = \int dx^d \, p(x^d) f_d(x^d, \beta_r^d) f_d(x^d, \beta_s^d) = c^d(\beta_r^d, \beta_s^d)$. Each C^d has dimension $N_d \times N_d$. The relationship between the functions c^d and the matrices C^d is analogous to the relationship between the function c and the matrix C: the function c^d encodes the covariance that comes from the dth factor for any two neurons, while the matrix C^d captures these values for the particular tuning curve parameters (i.e., neurons) sampled in the population.

The (m, n)th entry of C is the covariance between the mth and nth neurons, with parameter vectors $\boldsymbol{\alpha}_m$ and $\boldsymbol{\alpha}_n$ and can be expressed in terms of the C^ds:

$$
\begin{aligned}
C_{mn} = c(\boldsymbol{\alpha}_m, \boldsymbol{\alpha}_n) &= \prod_{d=1}^{D} c^d(\alpha_m^d, \alpha_n^d) \\
&= \prod_{d=1}^{D} c^d(\beta_{\Psi^d(m)}^d, \beta_{\Psi^d(n)}^d) \\
&= \prod_{d=1}^{D} C_{\Psi^d(m)\Psi^d(n)}^d \qquad (35)
\end{aligned}
$$

As described in Section S3.1, this functional form is simply the definition of the tensor product (as represented by the Kronecker product) and thus

$$
C = C^1 \otimes \cdots \otimes C^D. \qquad (36)
$$

Finally, we express the eigenvalues of C in terms of products of the eigenvalues of the C_d's. Let $\{\gamma_{p_d}^d, \boldsymbol{u_{p_d}^d}\}$ be the p_dth eigenvalue-eigenvector pair for each C^d. Note that

$$
\begin{aligned}
C\left(\boldsymbol{u_{p_1}^1} \otimes \cdots \otimes \boldsymbol{u_{p_D}^D}\right) &= \left(C^1 \otimes \cdots \otimes C^D\right)\left(\boldsymbol{u_{p_1}^1} \otimes \cdots \otimes \boldsymbol{u_{p_D}^D}\right) \\
&= \left(C^1 \boldsymbol{u_{p_1}^1}\right) \otimes \cdots \otimes \left(C^D \boldsymbol{u_{p_D}^D}\right) \\
&= \left(\gamma_{p_1}^1 \boldsymbol{u_{p_1}^1}\right) \otimes \cdots \otimes \left(\gamma_{p_D}^D \boldsymbol{u_{p_D}^D}\right) \\
&= \left(\gamma_{p_1}^1 \times \cdots \times \gamma_{p_D}^D\right)\left(\boldsymbol{u_{p_1}^1} \otimes \cdots \otimes \boldsymbol{u_{p_D}^D}\right)
\end{aligned} \qquad (37)
$$

Consequently, $\left(\gamma_{p_1}^1 \times \cdots \times \gamma_{p_D}^D\right)$ is an eigenvalue of C. All N eigenvalues of C can be constructed this way, as products of the eigenvalues of the factor C^d. Consequently, the eigenvalues of C are given by all possible products of the eigenvalues of the individual factors.

S3.3 The linear dimension of multiplicative models grows exponentially with intrinsic dimension

S3.3.1 Factors have same functional form

For simplicity, we first consider a multiplicative model where the tuning to each dimension (i.e., the functions f_d) takes the same functional form. Thus, each factor C^d is the same.

Let the eigenvalues of the covariance matrix for each factor be $\boldsymbol{\gamma} = \{\gamma_1, \ldots, \gamma_{N_D}\}$. Note that as we are considering fraction of variance explained, we can rescale $\{\gamma_1, \ldots, \gamma_{N_D}\}$ to sum to 1. The N eigenvalues of the overall covariance matrix C are given by all products of the form $\prod_d \gamma_{p_d}$, where each $p_d \in [1, \ldots, N_D]$.

To compute the linear dimension we reframe the problem as a problem in probability theory. Consider a set of D independent random variables Z_d, each distributed according to a categorical distribution with outcome probabilities $\{\gamma_1, \ldots, \gamma_{N_D}\}$. That is, Z_d takes values in $\{1, \ldots, N_d\}$ and $P(Z_d = k) = \gamma_k$. Moreover let the joint random variable $Z = (Z_1, \ldots, Z_D)$, where the Z_d's are independent. Thus the probability distribution of Z is the product of the distributions of the individual Z_d's.

Each eigenvalue of C is in one-to-one correspondence with an outcome of Z, and the value of the eigenvalue corresponds to the probability of that outcome. For example, the largest eigenvalue γ_1^D corresponds to the case where $Z_1 = \ldots = Z_D = 1$ (which happens with probability γ_1^D).

Finding the smallest set of eigenvalues whose sum is at least $1-\epsilon$ (i.e., the $L_{1-\epsilon}$ linear dimension) is equivalent to finding the smallest set of outcomes of Z whose probability is at least $1-\epsilon$. This subset is often referred to as an ϵ-high-probability set [4].

Standard results in information theory provide bounds on the size of this set[1] [4]. In particular, the asymptotic equipartition property (AEP) shows that as D increases, the number of elements in the high-probability set approaches $2^{DH(\boldsymbol{\gamma})}$, where $H(\boldsymbol{\gamma}) = -\sum_p \gamma_p \log_2 \gamma_p$ is the Shannon entropy of the distribution $\{\gamma_1, \ldots, \gamma_{N_d}\}$. Given that each element in the high-probability set corresponds to an eigenvalue of the covariance matrix C, the number of eigenvalues required and hence the $L_{1-\epsilon}$ dimension asymptotically grows as $2^{DH(\boldsymbol{\gamma})}$, thus increasing exponentially with D.

In the main text, Fig. 4b-g, we numerically verify that exponential scaling applies to tuning curves when factors have the same functional form.

S3.3.2 Factors have different functional forms

In the case of non-identical factors, let the eigenvalues of the covariance matrix for the dth factor be $\boldsymbol{\gamma^d} = \{\gamma_1^d, \ldots, \gamma_{N_d}^d\}$. As before, we can normalize so that $\sum_p \gamma_p^d = 1$. Also as before, we define a set of D independent categorical random variables $\{Z_1, \ldots, Z_D\}$, with $P(Z_d = k) = \gamma_k^d$, and observe that the eigenvalues of C correspond to the probabilities of the outcomes of $Z = (Z_1, \ldots, Z_D)$.

To apply the AEP to this setting, note that the AEP uses the weak law of large numbers to show that the average of log probability converges to its expectation value (i.e., the entropy) and this convergence applies to non-identical but independent random variables if variance is bounded (Chebyshev inequality). The corresponding variance here is $\sum_p \gamma_p^d \log_2^2(\gamma_p^d)$, the variance of log probability when the γ_p's are interpreted as probability values. Thus we assume that $\sum_p \gamma_p^d \log_2^2(\gamma_p^d) < C$ for some constant C. Note that this boundedness is a very mild condition—$\gamma_p \log^2(\gamma_p)$ is bounded by 0.127 on $[0, 1]$, so the only way for the sum to diverge as d increases is if the number of non-zero

[1] More specifically, they first show that most outcomes concentrate on a different subset called the *typical set* and then show that a high-probability set must have significant overlap with this typical set and thus be of about the same size.

eigenvalues for the dth factor and the spread of these eigenvalues both grow without bound as d increases.

Given that the variance of log probability is bounded, the AEP again holds, and the size of the high-probability set asymptotically approaches $2^{D\bar{H}}$, where $\bar{H} = \frac{1}{D}\sum_{d=1}^{D} H(\boldsymbol{\gamma^d})$ is the average entropy of the eigenvalue distribution of each factor.

Note that these results imply that, at least asymptotically, the linear dimension of the product of two factors or groups of factors is equal to the product of the linear dimensions of the factors or groups of factors.

In the main text, Fig. 4h-j, we numerically verify that exponential scaling applies to multiplicative population codes in which the factors are different. In Fig. S1, we also show results from a more general model-agnostic framework. For these simulations, rather than choosing specific functional forms for the factors (i.e., the f_d's in Eq. 33), we directly simulate eigenvalue distributions for the matrices C^d. Given that we can choose an overall normalization for eigenvalues (since ultimately we are interested in fraction of variance explained), we assume that the eigenvalues sum to 1 and thus form a probability distribution. This normalization allows us to generate candidate eigenvalue distributions using symmetric Dirichlet distributions, commonly used as a means to generate discrete distributions. The concentration parameter of the symmetric Dirichlet distribution determines how sparse the generated distributions are, and allows us to control the linear dimension of the individual factors. We use three different concentration parameters, moving from quite sparse to dense eigenvalue profiles for the single factors (shown in the first row of Fig. S1). We then compute the linear dimension of the tensor product and compare it to the theoretical lower bound, establishing exponential scaling.

S3.3.3 Finite size results and convergence to asymptotic scaling

While the results above provide asymptotic scaling, exponential scaling holds for small D as well, as we support in this section.

Non-asymptotic lower bound for identical factors For a non-asymptotic lower bound on the linear dimension with identical factors, consider the case where only two of the eigenvalues of the matrix C^d are nonzero. By normalizing to sum to 1, we can write these eigenvalues as $1-\gamma$ and γ, for some $\gamma \leq 0.5$. As for the multinomial case, the eigenvalues of C are the tensor product of $[1-\gamma, \gamma]$ taken D times, i.e. $\underbrace{[1-\gamma,\gamma] \otimes \cdots \otimes [1-\gamma,\gamma]}_{D\ times}$. In descending order of magnitude, there is 1 eigenvalue of magnitude $(1-\gamma)^D$, $\binom{D}{1}$ eigenvalues of magnitude $(1-\gamma)^{D-1}\gamma$, and so on, with $\binom{D}{k}$ eigenvalues of magnitude $(1-\gamma)^{D-k}\gamma^k$.

To lower bound the linear dimension, note that if K is such that

$$\sum_{k=0}^{K} \binom{D}{k} (1-\gamma)^{D-k}\gamma^k \leq (1-\epsilon), \tag{38}$$

then the linear dimension $L \geq \sum_{k=0}^{K}\binom{D}{k}$. Thus, we will first find such a K and then use it to lower bound the linear dimension L.

Consider a random variable Z distributed according to the binomial distribution with probability γ. That is, $X \sim Bin(D,\gamma)$. Note that $\sum_{k=0}^{K}\binom{D}{k}(1-\gamma)^{D-k}\gamma^k = P(X \leq K)$. The median of this binomial distribution is at least $\lfloor \gamma D \rfloor$. Thus if we choose $K = \lfloor \gamma D \rfloor - 1$ we have

$$\sum_{k=0}^{K} \binom{D}{k} (1-\gamma)^{D-k}\gamma^k < 0.5 < 0.95. \tag{39}$$

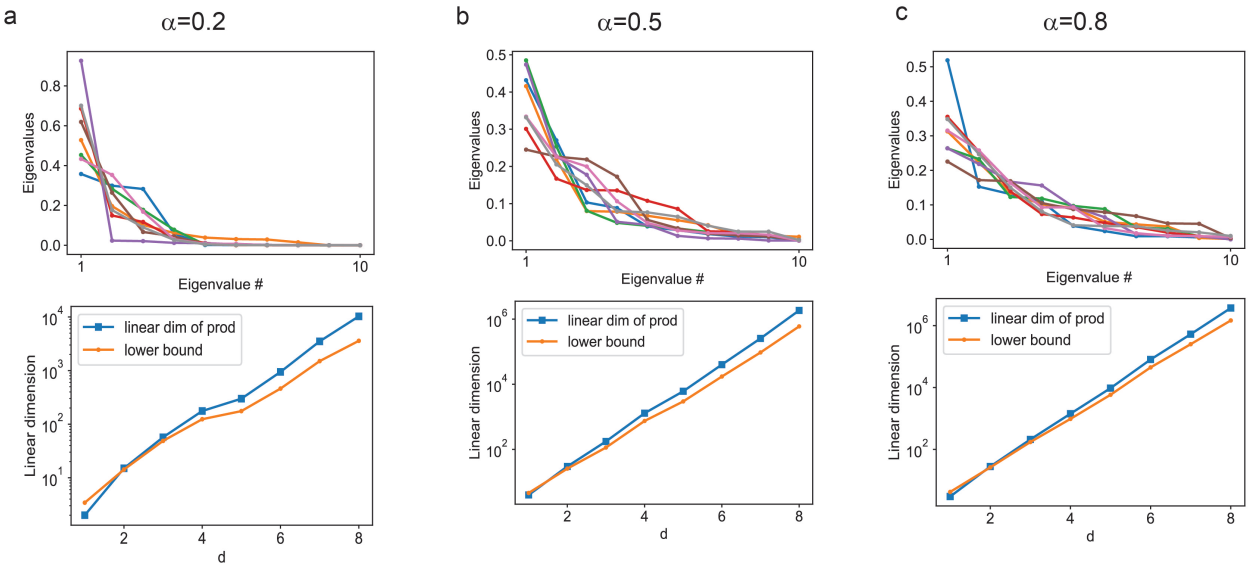

Figure S1: Linear dimension from different eigenvalue distributions for each dimension. (a) Top panel: Eigenvalue distributions (8 distributions, one for each dimension, of 10 eigenvalues each) were created from a symmetric Dirichlet distribution with concentration parameter $\alpha = 0.2$, yielding relatively sparse eigenvalue distributions. Bottom panel: linear dimension of tensor product of first D eigenvalue distributions along with lower bound. The lower bound is calculated as $2^{(\bar{H}-0.05)D}$, where $\bar{H}$ is the average entropy of the individual eigenvalue distributions. As in the main text, note that asymptotically $2^{(\bar{H}-\delta)D}$ is a lower bound for any δ, so the choice of 0.05 is for convenience but shows that exponential scaling applies for small D. (b) As in (a) but for $\alpha = 0.5$, (c) As in (a) but for $\alpha = 0.8$.

For simplicity, define γ' such that $\gamma' D = \lfloor \gamma D \rfloor$ and note that we can rewrite $K = \rho D$, where $\rho > \gamma - 2/D$ (or, using results bounding the distance of the mean to the median of a binomial, this can be improved to $\rho > \gamma - (1 + \ln(2))/D$). We can now lower bound L as $L \geq \sum_{k=0}^{\rho D} \binom{D}{k}$.

A standard bound on the sum of binomial coefficients [4] yields

$$\sum_{k=0}^{\rho D} \binom{D}{k} \geq \frac{1}{\sqrt{8D\rho(1-\rho)}} 2^{H(\rho)D} = \frac{1}{\sqrt{8\rho(1-\rho)}} 2^{(H_b(\rho) - \frac{log_2(D)}{2D})D} \tag{40}$$

where $H_b(\rho) = -\rho \log_2 \rho - (1-\rho)\log_2(1-\rho)$ is the binary entropy function.

Thus, except when D is small enough that $H(\rho) < \frac{\log_2(D)}{2D}$ the lower bound grows exponentially with D, with the exponent asymptotically approaching $H_b(\gamma)D$.

Empirical results In Fig. 4 and Fig. S1, we show an exponentially increasing lower bound of $2^{(\bar{H}-0.05)D}$. Note that this is an asymptotic lower bound (as is $2^{(\bar{H}-\delta)D}$ for any $\delta > 0$), and the 0.05 is chosen semi-arbitrarily. As can be seen for the plots, for $D \geq 2$ the linear dimension lies above this lower bound, showing that exponential scaling applies even for very small D.

Exponential scaling for linear dimension as measured by participation ratio An alternate measure of linear dimension is the participation ratio [5, 6]. If $\{\lambda_1, \ldots, \lambda_N\}$ are the eigenvalues of a covariance matrix, then the participation ratio (PR) is $\left(\sum_n \lambda_n\right)^2 / \sum_n \lambda_n^2$. We instead define linear dimension as the number of eigenvalues required to account for a certain fraction of total variance, as this is more easily interpretable in terms of data variance, and closely matches what is done in practice. However, PR is typically well-correlated with our definition of linear dimension [6], and is a much more theoretically tractable quantity. In particular, if the covariance matrix C is the tensor product of a set of components C^d (i.e., as in Eq. 36), then $PR(C) = \prod_d PR(C^d)$ and thus grows exponentially with D. To see this it is sufficient to consider two factors C^1 and C^2, with eigenvalues $\boldsymbol{\gamma^1} = \{\gamma_1^1, \ldots, \gamma_{N_1}^1\}$ and $\boldsymbol{\gamma^2} = \{\gamma_1^2, \ldots, \gamma_{N_2}^2\}$ respectively. The eigenvalues of the covariance matrix are all products of the form $\gamma_{p_1}^1 \gamma_{p_2}^2$. Then we have

$$PR(C) = \frac{\left(\sum_{p_1=1}^{N_1} \sum_{p_2=1}^{N_2} \gamma_{p_1}^1 \gamma_{p_2}^2\right)^2}{\sum_{p_1=1}^{N_1} \sum_{p_2=1}^{N_2} \left(\gamma_{p_1}^1 \gamma_{p_2}^2\right)^2} = \frac{\left(\sum_{p_1=1}^{N_1} \gamma_{p_1}^1\right)^2 \left(\sum_{p_2=1}^{N_2} \gamma_{p_2}^2\right)^2}{\sum_{p_1=1}^{N_1} \left(\gamma_{p_1}^1\right)^2 \sum_{p_2=1}^{N_2} \left(\gamma_{p_2}^2\right)^2} = PR(C^1)PR(C^2). \tag{41}$$

S4 Further numerical experiments

In this section we show via numerical simulations that results hold when assumptions are weakly violated. We use the canonical example of multidimensional Gaussian tuning as the test case throughout. For all plots in this section we use the 0.9-linear dimension (i.e., $\epsilon = 0.1$). In all plots, error bars (shown as shaded regions) extend from the 10th to 90th percentile of the data (and include an additional spread of 1 unit to account for the discreteness of linear dimension).

In Fig. S2, we numerically confirm the results of Section S1 and show that the linear dimensions of non-mean-subtracted and mean-subtracted data are very similar, and that subtracting 1 from the linear dimension of non-mean-subtracted data provides a lower bound on the linear dimension of mean-subtracted data.

The figures in the main text show periodic (circular) boundary conditions and tuning curve centers chosen to tile the space (i.e., located at the points of a lattice in the latent space). In Fig. S3 we show that similar results hold for non-periodic boundary conditions and when tuning curve centers are chosen randomly at uniform through the latent space.

The analyses for Gaussian tuning curves in Sections S2.3 and S2.5 require that in order for exponential scaling to hold, the tuning curves should not be "too wide". In Fig. S4 we show scaling for a variety of tuning curve widths, straddling the transition between exponential and sub-exponential scaling. As shown in the right inset, for exponential scaling to break down the tuning curves need to be very wide—wide enough that any single neuron responds to all values of the stimulus or latent variable.

In Fig. S5, we show how linear dimension behaves as a function of the number of neurons recorded for the multidimensional case (the one-dimensional case is included in Fig. 2 in the main text). These plots illustrate that if the number of neurons recorded is very low, then the linear dimension appears artificially low (note that the linear dimension is always less than or equal to the number of recorded neurons). However, it increases rapidly as more neurons are recorded until it reaches the true value.

In Fig. S6 we show that results for translation symmetric populations hold when tuning curves are only approximately shifted copies of each other rather than all having the same shape.

Finally, the results presented in the study for translation symmetric populations so far assume that tuning curve centers either tile the space (i.e., are located at the points of a lattice in the latent space) or are sampled randomly but uniformly through the space. However, there often exist privileged locations in stimulus or latent variable space that are encoded with a higher density of tuning curve centers. Examples include orientation tuning in area V1, where the horizontal and vertical directions have more tuned neurons [7], and place cells in the hippocampus, which cluster around reward and landmark locations [8]. To address this setting, in Fig. S7 we show simulated results from models with a mixture of tuning curves, consisting of a translation-symmetric background population (i.e., as shown in the main paper) and an additional population of neurons whose tuning curves are clustered around certain preferred locations (e.g., modeling cardinal directions in visual space or reward locations in a hippocampal place field map).

We consider two settings for the additional population:

1. In the first setting, shown in Fig. S7b and c, the added tuning curves have the same width as the background population—this situation would model, e.g., place fields clustering around reward or landmark locations. We find that the linear dimension is almost unchanged by these additional neurons. The preserved linear dimension is because as long as the background population covers the space the added neurons overlap with existing neurons. Thus adding more neurons at certain locations simply adds rows to the data matrix that can be well-approximated by linear combinations of existing rows and the rank of the data matrix does not change. Of course these additional neurons likely offer benefits in terms of faster decoding, noise reduction and so on.

2. In the second setting, shown in Fig. S7d and e, the added tuning curves have sharper tuning than the rest of the population, reflecting higher resolution encoding at certain locations. In this case, the added neurons effectively make the population code sparser and thus increase the linear dimension.

Thus, for both settings the linear dimension for the homogeneous case is a lower bound for the linear dimension of the inhomogeneous case.

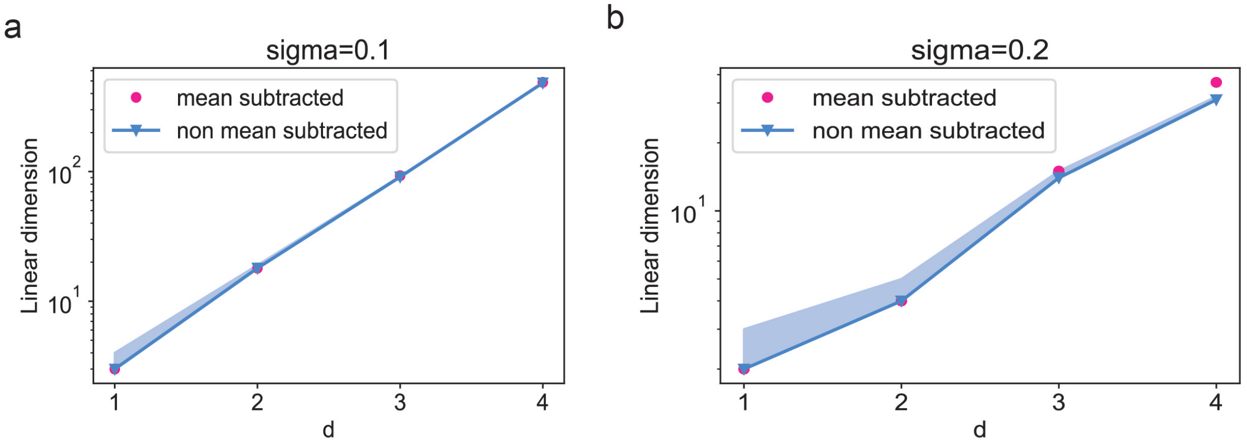

Figure S2: Linear dimension is similar between non-mean-subtracted and mean-subtracted-data. a) Multivariate Gaussian tuning with $\sigma = 0.1$. b) Multivariate Gaussian tuning with $\sigma = 0.2$.

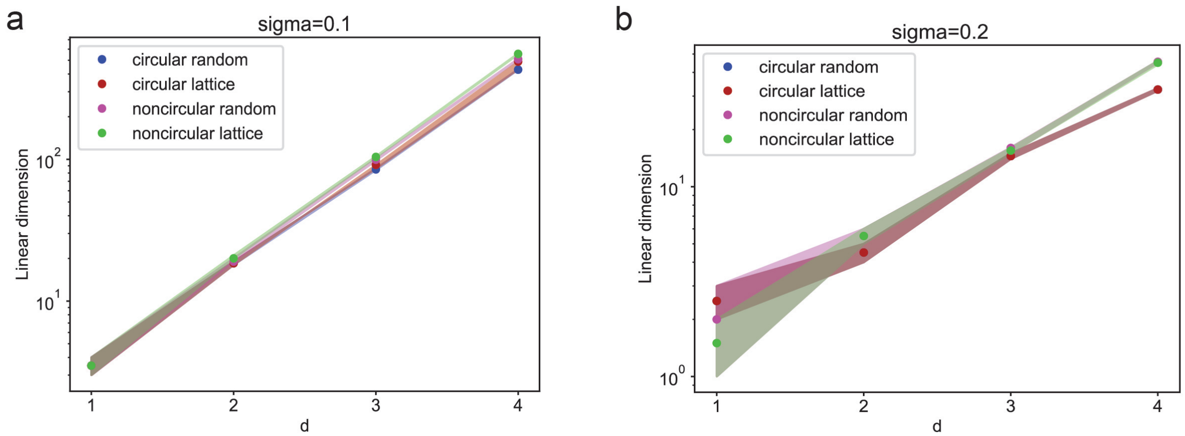

Figure S3: **Linear dimension of data from Gaussian tuning curves with different boundary conditions (b.c) and distribution of tuning centers.** Circular b.c and random tuning centers in $[0, 1]^D$ (blue). Circular b.c. and tuning centers on a lattice (maroon). Non-circular b.c and random tuning centers in $[0, 1]^D$ (magenta). Non-circular b.c. and lattice tuning centers (green). a) Linear dimension against D for $\sigma = 0.1$ b) Same as a) for $\sigma = 0.2$. Number of neurons used is as follows: d=1, 40; d=2, 400; d=3, 1000; d=4, 4096.

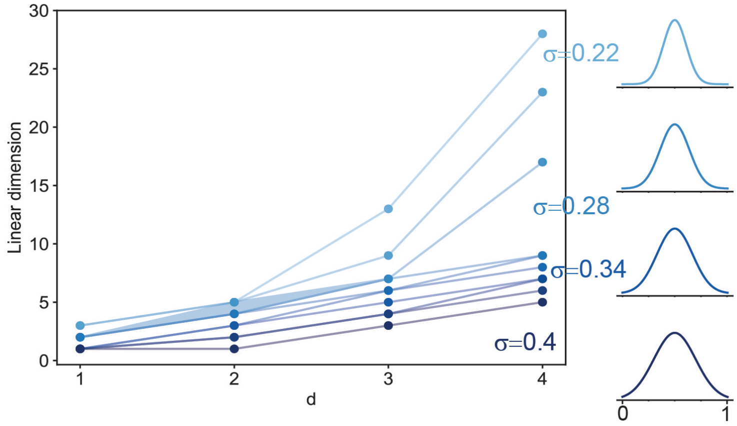

Figure S4: **Linear dimension for wider tuning curves** Linear dimension vs. D for wide tuning with σ between 0.22 to 0.4 in steps of 0.02. Corresponding Gaussians of widths $\sigma = 0.22, 0.28, 0.34, 0.4$ shown on the side. The exponential scaling transitions to subexponential scaling once tuning curves cover the entire range of the period [0,1].

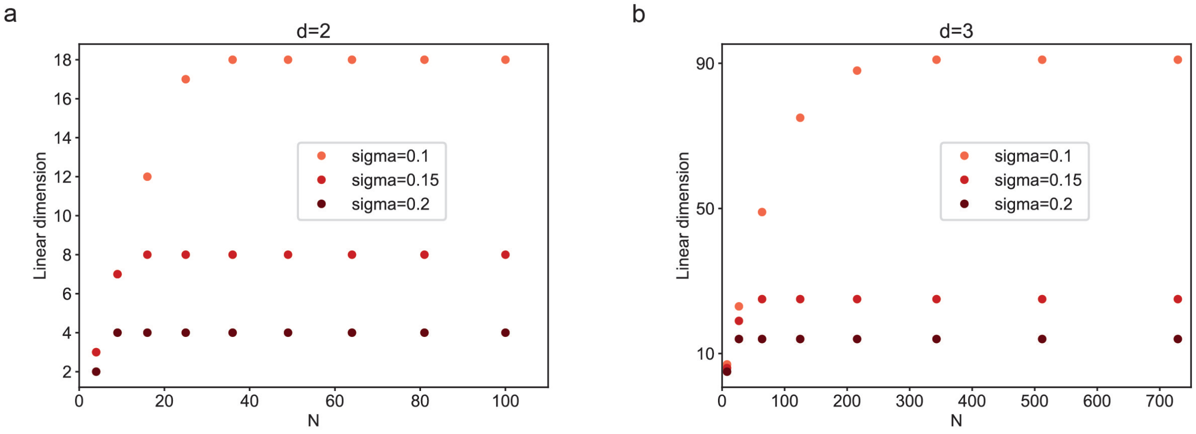

Figure S5: **Linear dimension as a function of the number of neurons (N).** a) Linear dimension against N for d=2. b) Linear dimension against N for d=3.

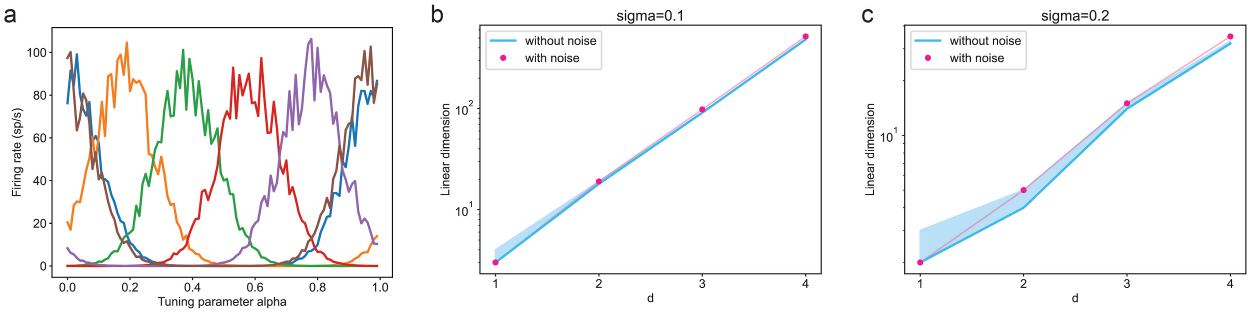

Figure S6: **Linear dimension for approximately translation symmetric tuning curves.** Each tuning curve was point wise multiplied by a random matrix of the same shape as the tuning curves whose elements were drawn uniformly from $[1-\epsilon, 1+\epsilon]$, with $\epsilon = 0.2$ (i.e., 20% multiplicative noise). The linear dimensions for data with noise lie within the 90% confidence interval of the linear dimensions of data without noise. a) Example of 7 neurons with circular Gaussian tuning curve with multiplied noise. b) Linear dimension against d for data from d dimensional Gaussian tuning curves multiplied by noise for $\sigma = 0.1$. c) Same as (b) for $\sigma = 0.2$.

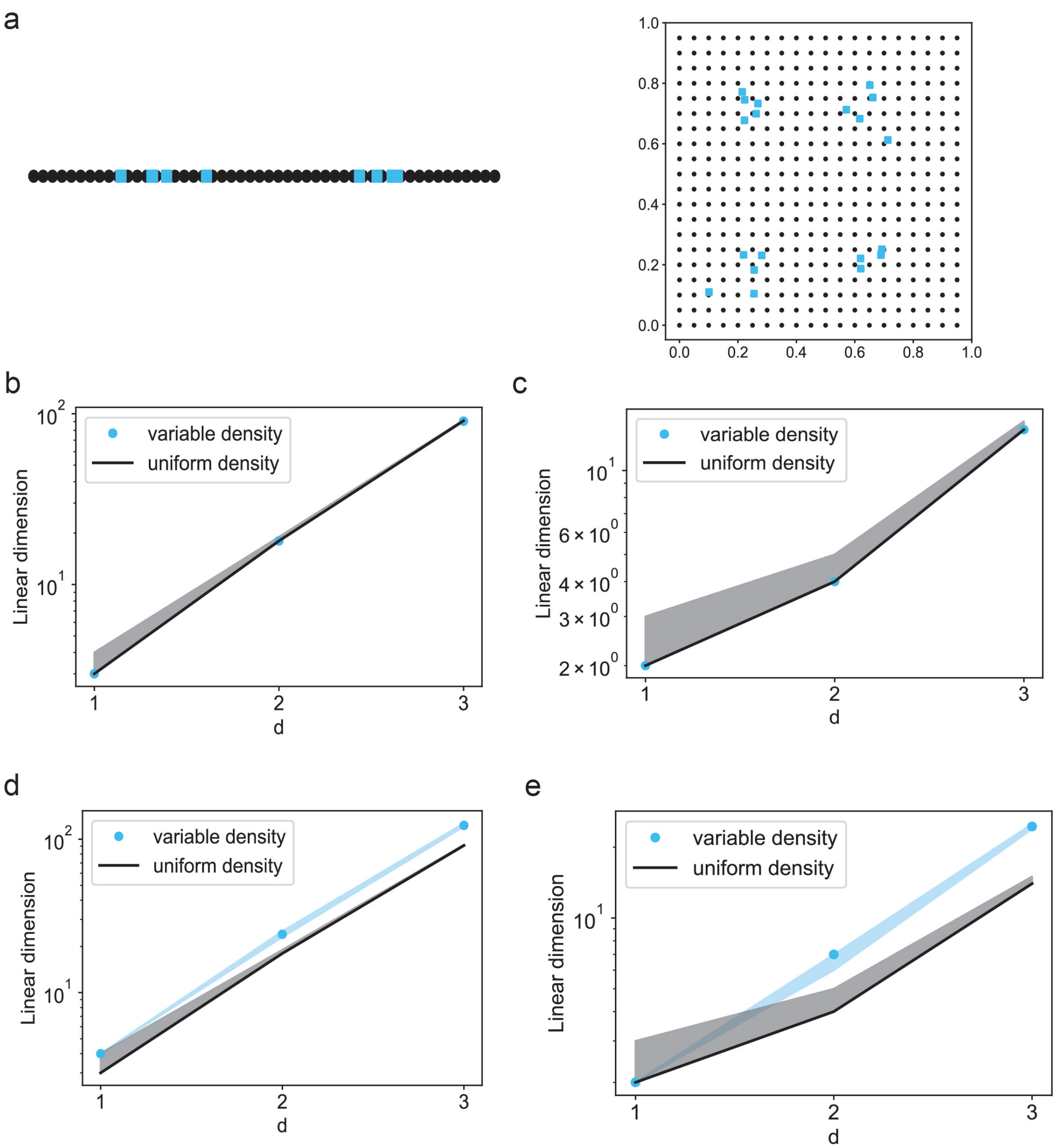

Figure S7: **Linear dimension of data from a background translation-symmetric population along with a higher density of neurons at special locations.** a) Schematic of the two populations, shown for 1D (left) and 2D (right). One population (black) has tuning centers that evenly cover the space; a second population (blue) has tuning centers that cluster around certain locations. (b) Both populations have tuning width of $\sigma = 0.1$ (c) As in (b) but for $\sigma = 0.2$. (d) Background population has tuning width of 0.1 while population clustered at special locations has sharper tuning width of 0.05. (e) As in (d) but tuning widths of 0.2 and 0.1.

References

1. Gray, R. On the asymptotic eigenvalue distribution of Toeplitz matrices. *IEEE Trans. Inf. Theory* **18,** 725–730 (1972).
2. Gray, R. M. Toeplitz and circulant matrices: A review. *Foundations and Trends in Communications and Information Theory* **2,** 155–239 (2006).
3. Wigderson, A. & Wigderson, Y. The uncertainty principle: variations on a theme. *Bulletin of the American Mathematical Society* **58,** 225–261 (2021).
4. Cover, T. M. & Thomas, J. A. *Elements of Information Theory, 2nd Edition* (Wiley-Interscience, 2006).
5. Abbott, L. F., Rajan, K. & Sompolinsky, H. *The Dynamic Brain: An Exploration of Neuronal Variability and Its Functional Significance* (eds Ding, M. & Glanzman, D.) 65–82 (Oxford University Press, Oxford, 2011).
6. Gao, P. *et al.* A theory of multineuronal dimensionality, dynamics and measurement. *bioRxiv,* 214262 (2017).
7. Ganguli, D. & Simoncelli, E. Implicit encoding of prior probabilities in optimal neural populations. *Adv. Neural Inf. Process. Syst.* **23** (2010).
8. Hollup, S. A., Molden, S., Donnett, J. G., Moser, M.-B. & Moser, E. I. Accumulation of hippocampal place fields at the goal location in an annular watermaze task. *J. Neurosci.* **21,** 1635–1644 (2001).
